# The Transcriptional Co-Repressor Myeloid Translocation Gene 16 Inhibits Glycolysis and Stimulates Mitochondrial Respiration

**DOI:** 10.1371/journal.pone.0068502

**Published:** 2013-07-01

**Authors:** Parveen Kumar, Vladimir V. Sharoyko, Peter Spégel, Urban Gullberg, Hindrik Mulder, Inge Olsson, Ram Ajore

**Affiliations:** 1 Department of Hematology, Lund University, Lund, Sweden; 2 Department of Clinical Sciences, Unit of Molecular Metabolism, Lund University Diabetes Centre, Malmö University Hospital, Malmö, Sweden; Southern Illinois University School of Medicine, United States of America

## Abstract

The myeloid translocation gene 16 product MTG16 is found in multiple transcription factor–containing complexes as a regulator of gene expression implicated in development and tumorigenesis. A stable Tet-On system for doxycycline–dependent expression of *MTG16* was established in B-lymphoblastoid Raji cells to unravel its molecular functions in transformed cells. A noticeable finding was that expression of certain genes involved in tumor cell metabolism including 6-phosphofructo-2-kinase/fructose-2,6-biphosphatase 3 and 4 (*PFKFB3* and *PFKFB4*), and pyruvate dehydrogenase kinase isoenzyme 1 (*PDK1*) was rapidly diminished when MTG16 was expressed. Furthermore, hypoxia–stimulated production of PFKFB3, PFKFB4 and PDK1 was inhibited by MTG16 expression. The genes in question encode key regulators of glycolysis and its coupling to mitochondrial metabolism and are commonly found to be overexpressed in transformed cells. The MTG16 Nervy Homology Region 2 (NHR2) oligomerization domain and the NHR3 protein–protein interaction domain were required intact for inhibition of *PFKFB3*, *PFKFB4* and *PDK1* expression to occur. Expression of MTG16 reduced glycolytic metabolism while mitochondrial respiration and formation of reactive oxygen species increased. The metabolic changes were paralleled by increased phosphorylation of mitogen–activated protein kinases, reduced levels of amino acids and inhibition of proliferation with a decreased fraction of cells in S-phase. Overall, our findings show that MTG16 can serve as a brake on glycolysis, a stimulator of mitochondrial respiration and an inhibitor of cell proliferation. Hence, elevation of MTG16 might have anti–tumor effect.

## Introduction

Myeloid translocation gene 16 (***MTG16***) (murine *ETO-2*) is one member of a remarkably conserved family of corepressors showing homology to *Nervy* in Drosophila [1]. Other family members in mammalian cells are ***ETO*** (Eight–TwentyOne) or MTG8 and MTG–related protein 1 (***MTGR1***). All family member genes are involved in chromosomal translocations of leukemia as fusion partners to the gene encoding the transcription factor AML1 [2], [3], [4], [5], [6]. MTG16 is found in multiple transcription factor–containing complexes as an important regulator of gene expression in development and tumorigenesis. Like other ETO homologue proteins, MTG16 only binds DNA in cooperation with site-specific transcription factors [7] competing out coactivators and causing repressed chromatin conformation by the action of recruited histone deacetylases (HDACs). MTG16 links a number of transcription factors to its chromatin silencing machinery [8], [9], [10], [11], [12]. Among the members of its family, MTG16 is the most highly expressed in primary hematopoietic cells confined especially to stem/progenitor, erythroid, megakaryocytic and B cells [13], [14]. Furthermore, murine MTG16 (ETO-2) may play a hematopoietic role by suppressing entry of stem cells into cell cycling, maintaining their quiescence [15]. In addition, a role for MTG16/ETO-2 has been suggested in controlling both erythropoiesis [10] and megakaryopoiesis [16]; during erythroid differentiation, ETO-2 is the silencing non-DNA binding component of a GATA-1-SCL/TAL1 complex. Moreover, results from targeted disruption of ETO-2 have suggested a role in cell fate decisions, proliferation and stress–dependent hematopoiesis [17]. Loss of function of *MTG16* through haploinsufficiency by allele disruption in the chromosomal translocation t(16;21) may contribute to leukemia, but a possible mechanism is concealed. In addition, MTG16 is reported to have tumor suppressor properties in solid tumors, for instance in breast cancer [18]. Aberrant *MTG16* epigenetic silencing has been reported in breast tumors [19]. To conclude, a wide range of studies indicates MTG16 to be a major corepressor in transcription factor complexes.

Differentiated cells rely heavily on mitochondrial oxidative phosphorylation to generate energy for homeostasis. Contrary to this, proliferating tumor cells, including leukemia cells, predominantly rely on glycolytic energy production and most glucose is converted to lactate. Thereby, mitochondrial respiration may be low even in oxygen–rich environments, a phenomenon termed the Warburg effect [20]. Hence, the metabolism of tumor cells, and other highly proliferating cells, is largely anabolic; this includes incorporation of nutrients into nucleotides, amino acids and lipids to synthesize macromolecules required for cell growth and proliferation [21]. In the present work, a striking finding from global gene expression analyses was that *MTG16* expression diminished the expression of genes for key glycolytic regulators involved in tumor cell metabolism. Furthermore, we report that elevation of MTG16 can lead to decreased glycolysis and stimulated mitochondrial respiration with increased formation of reactive oxygen species (ROS). This observation made us hypothesize that a glycolytic shift supporting cell growth and proliferation because of downregulation or loss of function of ETO homologue corepressors may promote cell transformation. Similarly, downregulation of ETO homologues may also support cell proliferation in non transformed cells. Our results demonstrated a metabolic switch from glycolysis to mitochondrial respiration, suggesting that *MTG16* could serve as a potential target for reversing the Warburg effect in transformed cells.

## Methods

### Cell Culture

The Burkitt's lymphoma human Raji cells [22], myelomonocytic U-937 cells [23], erytholeukemia HEL cells [24], erythroleukemia TF-1 cells [25], megakaryoblast MEG-01 cells [26], acute myeloid leukemia Kasumi-1 cells [27] and promyelocytic HL-60 cells [28] were grown in RPMI-1640 medium containing 10% Fetal Bovine Serum (FBS) (Gibco BRL, Life Technologies, Rockville, MD) and supplemented with 11.1 mM glucose. The TF-1 cells also received 20 ng/ml GM-CSF (R&D Systems Inc. Minneapolis, MN). Monkey kidney COS cells [29] were grown in DMEM medium containing 10% FBS. All cell lines were from ATCC.

### Transfection

An aliquot of 8×10^6^ Raji cells and plasmid in 0.4 ml of culture medium was electroporated by the Bio-Rad Electroporation Apparatus (Bio-Rad Laboratories, Hercules, CA) with electrical settings of 960 mF and 280 V. Antibiotic was added for selection of recombinant clones 48 h after electroporation. Individual clones growing in the presence of antibiotic were isolated, expanded into mass cultures and screened for expression.

### Generation of stable doxycycline inducible *MTG16* clones

The Tet-On 3G doxycycline inducible gene expression system (Clontech, Ozyme, Saint Quentin en Yulines, France) was used to control the expression of *MTG16* inserted under the TRE3G promoter (P_TRE3G_) in B-lymphoblastoid Raji cells. Culturing with the tetracycline analog doxycycline induces Tet-On 3 G transactivator binding to tet operator repeats within P_TRE3G_ followed by transcriptional activation of *MTG16*. Initially, Raji cells were transfected with the EF1α-Tet-3G plasmid (in which the EF1α promoter expresses the transactivator) by electroporation at 280V followed by expansion of individual clones growing under selection with 1 mg/ml geneticin. Stable Raji/Tet-3G clones were screened for expression of transactivator using the Promega Luciferase Assay System (Promega, Fitchburg, WI) after transient transfections with pTRE3G-Luc plasmid. Then, clones with high expression were electroporated with a linear hygromycin plasmid and pTRE3G-*MTG16* in which wild-type *MTG16* cDNA was incorporated downstream of Tet-regulated P_TRE3G_. Transfectants were selected in the presence of 0.5 mg/ml hygromycin. Induction of *MTG16* was accomplished by addition of doxycycline. Two out of 30 hygromycin–resistant clones displayed tightly regulated induction of MTG16 and were selected for further use.

Constructs with deletions of MTG16 Nervy Homology Region (NHR) 1 to 4 were also used for generation of stable doxycycline inducible clones in Raji cells in order to reveal functions associated with specific NHRs. Clones with high expression of EF1α-Tet-3G were electroporated with pTRE3G in which *MTG16*ΔNHR1 (amino acids 171–268 deleted), *MTG16*ΔNHR2 (amino acids 394–421 deleted), *MTG16*ΔNHR3 (amino acids 485–533 deleted) or *MTG*ΔNHR4 (amino acids 556–593 deleted) [30] had been incorporated downstream of Tet-regulated P_TRE3G_ in Raji cells.

### Microarray analysis

Total cellular RNA was extracted in triplicate from Raji/MTG16 Tet-On 3G cells after 8 h of incubation with or without 1 µg/ml doxycycline, using the RNeasy mini kit (Qiagen, Valencia, CA). The quality of RNA was examined by Bioanalyzer (Agilent, Santa Clara, CA). RevertAidTM (Fermentas Inc, Glen Burnie, MD) was used for synthesis of first strand cDNA from 1 µg RNA using random primers according to the manufacturers' instructions. Microarray analysis was performed using Affymetrix expression system at SCIBLU Genomics (BMC, Lund University, Lund, Sweden). Basic Affymetrix chip and experimental quality analyses were performed using the Expression Console Software v1.1.2 (Affymetrix, Santa Clara, CA). Robust Multi-array Analysis was performed for data normalization [31]. Statistical analysis was performed using the TMEV v4.0 software [32]. Significant genes were further validated with real time qPCR.

### Quantitative real time polymerase chain reaction (RT-qPCR)

Microarray results for selected genes were validated by RT-qPCR. Additionaly, RNA from Raji/Tet3G clones not transfected with *MTG16* was analyzed after incubation with doxycycline to rule out off-target effects. After isolation, RNA was incubated with DNase I, #EN0521 (Fermentas Inc, Glen Burnie, MD) for 30 min at 37oC. Then cDNA was synthesized using omniscript RT kit #20511 (Qiagen, Valencia, CA). The RT-qPCR reaction contained 7.5 µl 2×MAXIMA SYBR mix (Fermentas Inc, Glen Burnie, MD), 0.6 μmoles (0.6 µl) of each primer, 2 µl cDNA template and water to a final volume of 15 µl. PCR parameters were: 50°C for 2 min, 95°C for 10 min, 40×(95°C for 15 sec, 60°C for 30 sec and 72°C for 30 sec). Primers were designed as shown in supplementary Table S1. Human *18 S rRNA* and *GAPDH* were used as references. Relative quantification values were expressed using the ΔΔCt method normalized to the reference genes and related to the expression of the controls [33]. Normalization: ΔCt  =  Ct (sample) – Ct (geomean of Ct of GAPDH and 18 S rRNA). ΔΔCt  =  ΔCt (sample) - ΔCt (control). Relative quantification  = 2**^−ΔΔCt^**

### Western blotting

Western blotting was performed essentially as previously described [14], using the following antibodies: Polyclonal anti-MTG reactive with all ETO homologues [34]; rabbit polyclonal anti-histone H3 CHIP grade (# ab1791) (Abcam, Cambridge, UK); rabbit polyclonal anti-PFKFB4 (#PAB4031) (Abnova, Suffolk, UK); mouse monoclonal anti-p-c-jun (KM-1) (# sc-822), rabbit polyclonal anti-c-jun (H-79) (#sc-1694) (Santa Cruz, CA); rabbit monoclonal anti-PDHK1 (C47H1) (#3820), rabbit monoclonal anti-phospho–p44/42 MAPK (Erk1/2) (Thr 202/Tyr 204) (#4370), rabbit polyclonal anti-p38 MAPK (#9212), rabbit monoclonal anti-phospho p38 MAPK (Thr180/Tyr182) (D3F9) (#4511) (Cell Signalling, Danvers, MA).

### Glucose utilization assay

The rate of [^3^H]OH production from [5-^3^H]glucose was examined as previously described [35]. Glucose utilization was calculated as [{[^3^H]OH formed (CPM/min)}/{(specific radioactivity of D-[5-^3^H]glucose (CPM/min/pmol)}].

### Lactate assay

Cells were recovered by centrifugation and incubated in full medium for 4 h. Then the supernatant was collected and used for assay of released lactate by the Lactate assay kit # K607-100 from Biovision (Mountain View, CA).

### Respiration

The oxygen consumption rate (OCR) was measured by the Extracellular flux analyzer XF24 (Seahorse Bioscience, Houston, TX,) as previously described [35]. The assay medium contained 114 mM NaCl, 4.7 mM KCl, 1.2 mM KH_2_PO4, 1.16 mM MgSO_4_, 20 mM HEPES, 2.5 mM CaCl_2_, 0.2% bovine serum albumin, pH 7.2, and 11.1 mM glucose. The XF24 24-well plate was coated with 100 ug/ml poly-D-lysine (Millipore, Billerica,MA) at 37°C for 2 h prior to seeding of 250,000 cells/well (0.32-cm^2^ growth area) in 500 µl of RPMI 1640 medium. Cells were incubated for 1.5 h at 37°C in a humidified atmosphere of 95% air and 5% CO_2_. Then, the RPMI 1640 medium was removed and replaced by 750 µl assay medium. The adherent cells were preincubated for 30 min at 37°C in air after which respiration was measured in 11.1 mM glucose. ATP synthase was inhibited by injection of 4 µg/ml oligomycin, to discriminate between the ATP-linked respiration (oligomycin-sensitive respiration) and the proton leak. Then, the maximal respiratory capacity was determined after injection of 4 µM carbonyl cyanide-p-trifluoromethoxyphenylhydrazone (FCCP), which is a mitochondrial uncoupler of oxidative phosphorylation. Finally, 1 µM rotenone was injected to block transfer of electrons from complex I to ubiquinone.

### Reactive Oxygen Species (ROS) assay

ROS was monitored using the fluorescent probe dichlorofluorescein diacetate (DCFHDA) [36]. An aliquot of 500,000 cells in PBS was stained with 20 µM DCFHDA for 30 min at 37°C, washed and resuspended in PBS. Stained cells were transferred to a black plate as 100,000 cells/50 µl/well. Unstained cells were used as blanks. Readings were taken in a fluorescent plate reader TECAN Infinite M200 by setting excitation wavelength at 485 nm and emission wavelength at 535 nm.

### NADPH Assay

NADPH was measured with the NADP^+^/NADPH quantification kit #K347-100 from Biovision (Mountain View, CA) and expressed as pmol/10^6^ cells.

### Glutathione Assay

Total glutathione, reduced glutathione (GSH) and oxidized glutathione (GSSG) were individually measured in cell homogenates using the glutathione assay kit #K347-100 from Biovision (Mountain View, CA).

### Metabolite profiling

Metabolite profiling was performed as previously described [37]. Raji/MTG16 Tet-On 3G cells were incubated for 48 h without (control) and with 20 ng/ml doxycycline (to induce MTG16). Cells were then incubated for 2 h in HBSS containing 11.1 mM glucose followed by washing once in ice-cold PBS prior to quenching of the metabolism by the addition of 200 µl ice-cold water. A set of stable isotope-labelled internal standards was added and protein precipitated by 80% (v/v) methanol. Metabolites were extracted and evaporated to dryness. Prior to analysis by gas chromatography/mass spectrometry, metabolites were methoximated and trimethylsilylated. Samples were analyzed on an Agilent 6890N gas chromatograph (Agilent, Atlanta, GA) equipped with a 30 m DB-5MS column (J&W Scientific, Folsome, CA) and connected to a Leco Pegasus III electron impact time-of-flight mass spectrometer (Leco Corp., St Joseph, MI). Results on relative metabolite levels were acquired in ChromaTof (Leco Corp) and further processed in scripts developed in MATLAB™ software 2006b (Mathworks, Natick, MA) [38]. Identification of metabolites was based on database searches of mass spectra and retention indexes. Data were normalized to the internal standards [39] and to the protein contents measured using the BCA assay. An overview of data was generated by orthogonal projections to latent structures discriminant analysis (OPLS-DA) in Simca P+12.0 (Umetrics, Umeå, Sweden).

### Cell cycle analysis

Flow cytometric determinations of DNA content [40] were performed by FACS calibur (Becton-Dickinson, Franklin Lakes, NJ). The fraction of the cells in the G0-G1, S and G2-M was analysed using the Flowjo™ software (TreeStar, Ashland, OR).

### Statistical analysis

The significance of difference between samples was determined by the unpaired Student's *t* tests or the one- or two-way ANOVA followed by post hoc tests using the Graphpad Prism version 5.0a Software (GraphPad Software, Inc., CA), unless stated differently. Single and triple asterisks represent *P<0.05* and *P<0.0001*, respectively. Data are presented as means±SEM.

## Results

### Inducible expression of *MTG16* with a Tet-On system

**Figure 1:**
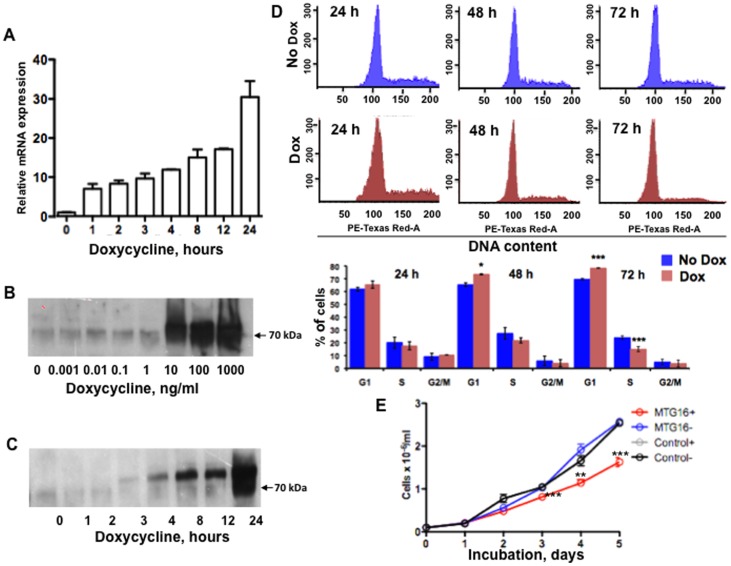
Doxycycline-induced expression of MTG16 in Raji/MTG16 Tet-On 3G cells. The Tet-On 3G doxycycline (DOX)–dependent gene expression system was used to regulate the expression of *MTG16* inserted under the control of a TRE3G promoter in B-lymphoblastoid Raji target cells (Raji/MTG16 Tet-On 3G cells). **A**. *MTG16* transcript induction with doxycycline. *MTG16* mRNA is produced within 1 h of incubation with 20 ng/ml doxycycline as shown by RT-qPCR. **B**. Dose–response relationship for doxycycline induction of MTG16. MTG16 Tet-On 3G cells were incubated with various concentrations of doxycycline for 24 h and cell lysates were probed with anti-MTG by Western blot analysis for protein detection. Close to maximal MTG16 expression was obtained with 10 ng/ml of doxycycline. **C**. Time course for doxycycline induction of MTG16. Cells were incubated with 10 ng/ml doxycycline for various time periods and cell lysates were probed with anti-MTG by Western blot analysis. MTG16 protein was detected within 3 to 4 h. **D**. Cell cycle analysis of doxycycline–induced cells. Cells were incubated without (no DOX, blue) or with 20 ng/ml doxycycline (DOX, red) at 2×10^5^/ml up to 72 h and cell cycle analyses were performed by flow cytometry at intervals indicated. Representative cell cycle analyses are shown. Cell cycle distribution is also shown as percentage of cells in each phase. **E**. Inhibition of proliferation in doxycycline–induced *MTG16* Tet-On cells. Proliferation curves are shown for *MTG16* Tet-On cells incubated without doxycycline (blue), *MTG16* Tet-On cells incubated with 20 ng/ml doxycycline (red) and control Raji cells expressing both EF1α-Tet3G and empty pTRE3G (without *MTG16*) incubated with 20 ng/ml doxycycline (black). MTG16 Tet-On cells incubated with doxycycline exhibited decreased proliferation compared to control cells. Cell viability was approximately 90% in all experiments and data are given for viable cells. Data are represented as means±SEM for (A) n = 3, (D) n = 3 and (E) n = 3 and were compared by the two-way ANOVA followed by Bonferroni (D) or the Tukey's multiple comparison post-hoc tests (E) (*p<0.05; ***p<0.001). Experiments depicted in B, C and flow cytometry experiments in D were repeated at least three times and representative results are shown.

We decided to use a doxycycline–regulated Tet-On time– and dose–dependent gene expression system [41] to achieve controlled expression of *MTG16* with an overall aim of finding concurrent changes in global gene expression and functions of MTG16. In efforts to create such a system we noted that MTG16 Tet-On positive clones established from hematopoietic cell lines often were unstable and reverted to wildtype. However, a stable MTG16 Tet-On system was successfully established in B-lymphoblastoid Raji cells. This system made it possible to unravel molecular functions of MTG16 that may be relevant to the function of this corepressor in transformed cells. A time course study following incubation with doxycycline showed elevated MTG16 mRNA within 1 h after induction (Figure 1A). MTG16 protein was detected at very low concentrations of doxycycline; maximal levels were observed at 10 ng/ml doxycycline (Figure 1B). MTG16 protein occurred between 3 to 4 h after induction (Figure 1C) and could be detected for at least 72 h (data not shown). The virtual lack of MTG16 expression in Raji/MTG16 Tet-On 3G cells exposed to doxycycline during 0 to 2 h indicates a low level of transgene leakiness. Unspecific effects of doxycycline are unlikely at the 10–20 ng/ml concentrations used. Thus, the Raji/MTG16 Tet-On 3G cell system is very sensitive to doxycycline, lacks background expression of the transgene and shows regulated expression.

Flow cytometry analysis showed that the cell cycle activity declined between 24 and 48 h of incubation with doxycycline (Figure 1D). Between 48 and 72 h a significant increase was observed of cells in the G0/G1 phase concomitant with a significantly decreased population of cells at the S phase (Figure 1D). The cell proliferation rate was decreased after MTG16 induction with doxycycline (Figure 1E). The inhibition of proliferation corresponded to the decrease in S-phase shown in Figure 1D; significant inhibition was observed after 72 h. Control cells doubly transfected with EF1α-Tet-3G and empty pTRE3G (without *MTG16*) showed no inhibition of proliferation upon incubation with doxycycline (Figure 1E). This indicates a lack of unspecific effects. Furthermore, no decrease in cell viability or increase in apoptosis was seen during doxycycline–induced MTG16 production (Figure S1C).

### Expression of 6-phosphofructo-2-kinase/fructose-2,6-biphosphatase 3 (*PFKFB3*), *PFKFB4* and pyruvate dehydrogenase kinase isoenzyme 1 (*PDK1*) was diminished when MTG16 was expressed

**Figure 2:**
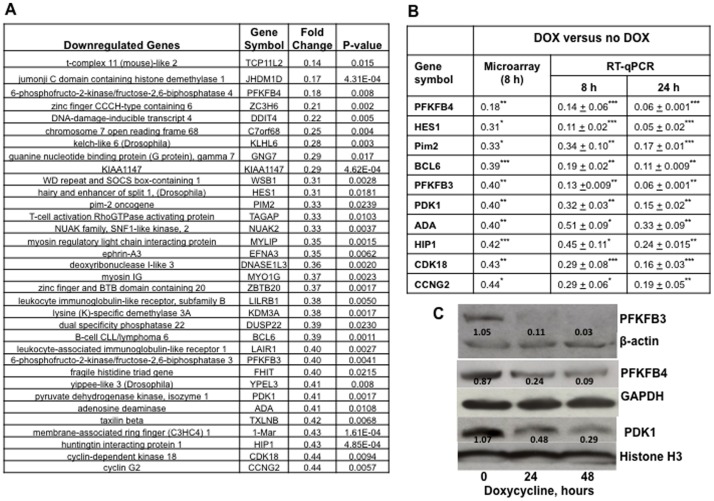
Doxycycline–induced expression of MTG16 altered gene expression. Gene expression profiling of mRNA was performed using Affymetrix human gene cDNA microarray. Aliquots of mRNA were isolated from Raji/MTG16 Tet-On 3G cells incubated without doxycycline as a control or with 1 µg/ml doxycycline 8 h for induction of *MTG16*. Biological replicates of cells were collected in triplicates on different days. The significant difference of gene expression between doxycycline _plus_ and doxycycline _minus_ cells was calculated. **A**. Genes whose expression was most downregulated by doxycycline. **B**. Validation of results from microarray with RT-qPCR for selected doxycycline–downregulated genes. RNA for RT-qPCR was from cells incubated 8 and 24 h without or with doxycycline (DOX) and data are represented as means±SEM for n = 3 and compared by the one-way ANOVA followed by the Dunnett's post-hoc test (*p<0.05; ***p<0.001). **C**. Quantification of PFKFB3, PFKFB4, PDK1, β-actin and Histone H3 protein by Western blot analysis. The intensity of the bands was quantified by densitometry and expressed as ratios to β-actin or Histone H3. Results are shown for cells incubated with 20 ng/ml doxycycline for 0, 24 and 48 h. The Western blotting experiments were repeated at least three times and representative results are shown. The levels of PFKFB3, PFKFB4 and PDK1 were decreased by doxycycline induction of MTG16.

To identify genes regulated by MTG16, we used a cDNA array to compare RNA expression between Raji/MTG16 Tet-On 3G cells incubated without doxycycline and cells incubated 8 h with doxycycline (Figure 2A, Table S2) (GEO, accession number GSE 42682).

We observed diminished expression of inhibitor of DNA binding 2 (*ID2*); this is in agreement with the corresponding upregulation of this gene in *Mtg16*–null lineage stem cells [15]. We found diminished expression of hairy and enhancer of split1 (*HES1*); this is in agreement with MTG16 being a co-repressor for the transcription of *HES1* [12]. As expected, diminished expression of B-cell lymphoma 6 protein (*BCL6*) was also observed [42].

Furthermore, expression of *PFKFB3*, *PFKFB4* and *PDK1* was decreased by MTG16-expression (Figure 2A); these genes are key regulators of glucose metabolism in tumor cells. The microarray data for genes whose expression was altered by MTG16-expression (e.g. regulators of glycolysis) were validated by RT-qPCR (Figure 2B).

The mRNA levels of *PFKFB3*, *PFKFB4* and *PDK1* were significantly downregulated within 4 h of *MTG16*–induction with 20 ng/ml doxycycline and remained reduced for 24 h (Figure S1A). Expression of the pyruvate kinase isoenzyme M2 gene (*PKM2*) [43], however, was increased by MTG16 (Figure S1A). Furthermore, gene expression data from control cells expressing Tet transactivator only, showed no downregulation of *PFKFB3*, *PFKFB4* or *PDK1* upon incubation with doxycycline (Figure S1B). Thus, inhibited gene expression is associated with elevated MTG16 expression and not by a Tet-transactivator effect. Western blot analysis confirmed diminished expression of PFKFB3, PFKFB4 and PDK1 at the protein level (Figure 2C). In conclusion, expression of genes essential for a positive regulation of glycolytic rate and negative regulation of oxidative phosphorylation was diminished by MTG16. Such regulation would be anticipated to account for enhanced mitochondrial and aerobic metabolism.

To determine whether doxycycline–induced *MTG16* expression in Raji/MTG16 Tet-On 3G cells was higher than endogeneous expression in hematopoietic cells a comparison was made with *MTG16* expression in various hematopoietic cell lines and CD34+ normal hematopoietic progenitor cells. The *MTG16* mRNA level of doxycycline–induced RAji/MTG16 cells was found to be in the same range as that of CD34+ cells or erythroid (HEL and TF-1) and megakaryocytic (MEG-01) cell lines (Figure S2). CD34+ cells show the highest *MTG16* mRNA expression among human primary hematopoietic cells [14]. Other hematopoietic cell lines examined, e.g. wild type Raji, promyelocytic HL-60, and t(8;21) Kasumi cells showed low levels or absence of *MTG16* transcripts.

### MTG16 diminished hypoxia–induced expression of *PFKFB3*, *PFKFB4* and *PDK1*

**Figure 3:**
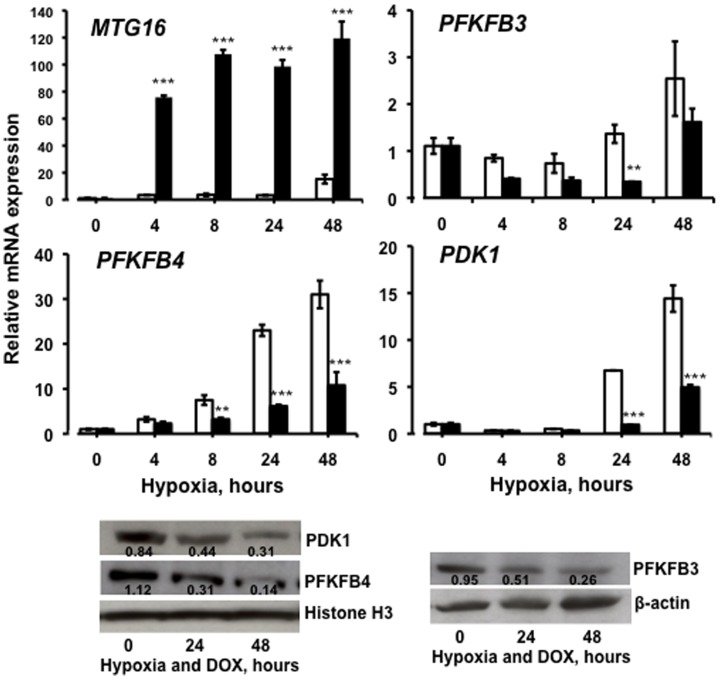
MTG16 inhibition of *PFKFB3, PFKFB4* and *PDK1* during hypoxia. The time course is shown for transcriptional expression of *MTG16*, *PFKFB3, PFKFB4*, and *PDK1* in Raji/MTG16 Tet-On 3G cells during incubation without doxycycline (open bars) or 20 ng/ml doxycycline (closed bars) under hypoxic conditions (4% O_2_). Upregulation of *MTG16* expression by doxycycline was achieved also during hypoxia. Doxycycline induction of MTG16 diminished the hypoxia induction of *PFKFB3, PFKFB4* and *PDK1* expression. For protein detection, cell lysates were probed with antibodies to PFKFB3, PFKFB4, PDK1, β-actin and Histone H3 by Western blot analysis. The intensity of the bands was quantified by densitometry and expressed as ratios to β-actin or Histone H3. Results are shown after incubation with 20 ng/ml doxycycline for 0, 24 and 48 h during hypoxia. The Western blotting experiments were repeated at least three times and representative results are shown. The level of PFKFB4 and PDK1 was decreased by doxycycline–induction of MTG16. Data are represented as means±SEM for n = 3 and were compared by the two-way ANOVA followed by the Bonferroni post-hoc test (*p<0.05; ***p<0.001).

Expression of the MTG16 inhibited genes *PFKFB3, PFKFB4* and *PDK1* are also regulated by hypoxia–inducible-factor-1 (HIF-1) [44], [45], [46]. Therefore, we examined whether transcription of these genes could be inhibited by MTG16 also at hypoxic conditions. To this end, cells were incubated in 4% O_2_ with 20 ng/ml doxycycline up to 48 h to induce expression of *MTG16* during hypoxia. Indeed, marked upregulation of *MTG16* transcripts was achieved by doxycycline also during hypoxia (Figure 3). Cells incubated without doxycycline showed substantial hypoxia–dependent upregulation of *PFKFB4* and *PDK1* with time whereas *PFKFB3* was upregulated only 2.5-fold at 48 h (Figure 3). Cells incubated with doxycycline showed inhibited transcriptional expression of *PFKFB4* and *PDK1* also during hypoxia (Figure 3). However, transcriptional inhibition of PFKFB3 was insignificant during hypoxia. Nevertheless, PFKFB3, PFKFB4 and PDK1 were significantly inhibited at the protein level (Figure 3). Taken together, our results confirm that MTG16 decreased the production of PFKFB3, PFKFB4 and PDK1 also under hypoxic conditions.

### Nervy Homology Region (NHR) 2 and 3 were required for MTG16–mediated inhibition of *PFKFB3*, *PFKFB4* and *PDK1*

**Figure 4:**
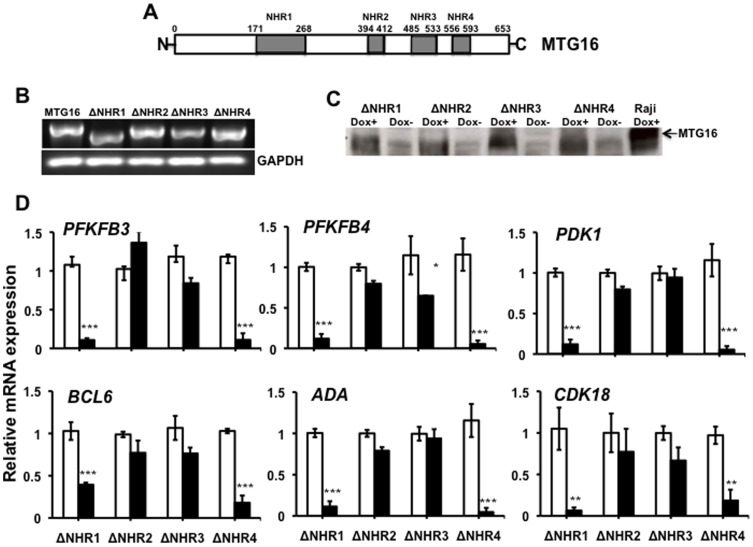
Requirement of Nervy Homology Region (NHR) 2 and 3 for MTG16–mediated gene expression inhibition. The Tet-On 3G doxycycline (DOX)–dependent gene expression system was used to regulate the expression of *MTG16* NHR1-4 deletions inserted under the control of a TRE3G promoter in B-lymphoblastoid Raji target cells. **A**. Scheme of MTG16 with NHRs indicated. Deletions of the individual NHRs indicated created *MTG16*ΔNHR1, *MTG16*ΔNHR2, *MTG16*ΔNHR3 and *MTG16*ΔNHR4. **B**. Expression of mRNA for *MTG16* NHR deletion 1-4 and full-length *MTG16*. Cells were incubated with 20 ng/ml doxycycline for 24 h to induce gene expression before isolation of RNA and examination with PCR as described in Methods. The mRNA of different NHR deletions showed an expected size. **C**. Doxycycline (Dox) induction of MTG16 NHR1-4 deletions. Cells were incubated with 20 ng/ml doxycycline for 24 h and MTG16 in cell lysates was probed by Western blot analysis. All MTG16 NHR deletions showed expected lower size than full–length MTG16 from doxycycline–induced Raji/MTG16 Tet-On 3G cells (Raji Dox+). The polyclonal anti-MTG antibody used in Western blotting was raised against amino acids 31–250 of MTG8 [41] and recognizes wildtype MTG16 as well as the deletion mutants used. **D**. Effect of MTG16 NHR1-4 deletions on RNA expression of selected genes the expression of which was inhibited by MTG16. Cells were incubated without doxycycline (open bars) or with 20 ng/ml doxycycline (closed bars) for 24 h followed by examination of gene expression by RT-qPCR. Deletion of NHR2 or 3 abolished MTG16–mediated gene repression. Data in C are represented as means±SEM for n = 3 and were compared by the two-way ANOVA followed by the Bonferroni post-hoc test (*p<0.05; ***p<0.001).

As recurrent mutations of *MTG16* have been found in human cancers at conserved NHRs [47], [48], we examined whether specific NHRs were required intact for changing gene expression. To this end, the individual NHRs were deleted to create *MTG16*ΔNHR1, *MTG16*ΔNHR2, *MTG16*ΔNHR3 and *MTG16*ΔNHR4. Deletion constructs were verified by sequencing. The Tet-On system was used to generate stable doxycycline inducible Raji cell clones of all the NHR deletion constructs. PCR results were consistent with expected size of mRNA for the various NHR deletions (Figure 4B). Timing of induction showed synthesis of MTG16 NHR1-4 deletion protein within 8 h (Figure 4C). Deletion of NHR2 or 3 abolished changes in expression of *PFKFB3*, *PFKFB4*, *PDK1* and the other examined MTG16-inhibited genes (Figure 4D). In contrast, deletion of NHR1 or NHR4 still permitted diminished expression of examined genes (Figure 4D). Thus, intact NHR2 and NHR3, but not NHR1 and NHR4, are essential for the MTG16–mediated inhibition of the genes investigated.

### MTG16 decreased glycolysis and stimulated mitochondrial respiration

**Figure 5:**
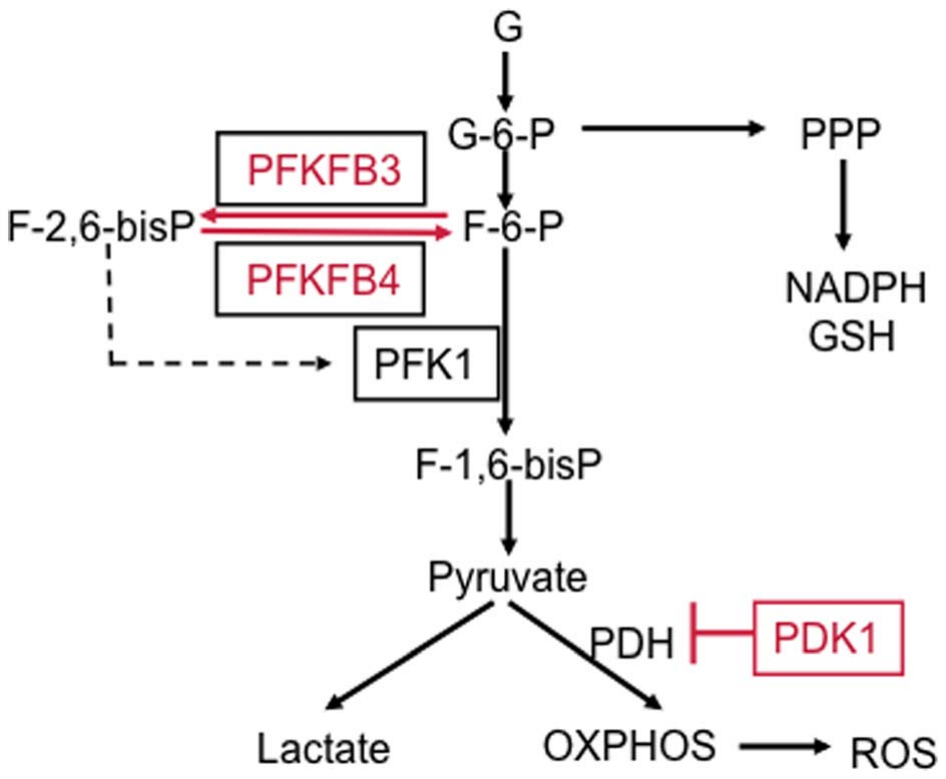
Scheme illustrating the impact of MTG16 provoked downregulation of PFKFB3, PFKFB4 and PDK1 on glucose metabolism. PFKFB3 exhibits a strong kinase to bisphosphatase activity ratio promoting production of F-2,6-bisP, which activates phosphofructokinase (PFK1), a key stimulator of glycolysis. PFKB4 may exhibit a higher bis-phosphatase than kinase activity promoting glucose shunting towards the pentose phosphate pathway (PPP). PDK1 is a negative regulator of pyruvate dehydrogenase (PDH) leading to decreased mitochondrial pyruvate import and resulting in reduced oxidative phosphorylation (OXPHOS).

The MTG16–induced diminished expression of genes of key enzymes in glucose metabolism, *PFKFB3*, *PFKFB4* and *PDK1*, suggested concurrent effects on cell metabolism. The scheme in Figure 5 illustrates functions of PFKFB3, PFKFB4 and PDK1 in cell metabolism. The bifunctional kinase and phosphatase PFKFB enzyme family controls the level of fructose-2,6-bisphosphate, which allosterically activates phosphofructokinase 1 (PFK1), the key regulator of glycolysis [49]. PFKFB3, frequently elevated in human tumors [50], exhibits a strong kinase to bisphosphatase activity ratio, promoting glycolytic activity. On the other hand, PFKFB4 may exhibit a higher bisphosphatase than kinase activity [51], promoting a higher glucose flux in the pentose phosphate pathway (PPP). PFKFB4 may also exhibit a nearly equal kinase to bisphosphatase activity [52]. The pyruvate dehydrogenase (PDH) complex converts glucose–derived pyruvate into acetyl-CoA in mitochondria. Repression of *PDK1*, a negative regulator of PDH, would lead to stimulation of the citric acid cycle and increased oxidative phosphorylation [53]. Thus, decreased PFKFB3 activity predicts diminished glycolysis and decreased PFKFB4 activity attenuated glucose flux through the PPP and/or inhibition of glycolysis. Decreased PDK1 activity predicts activation of mitochondrial respiration.

**Figure 6:**
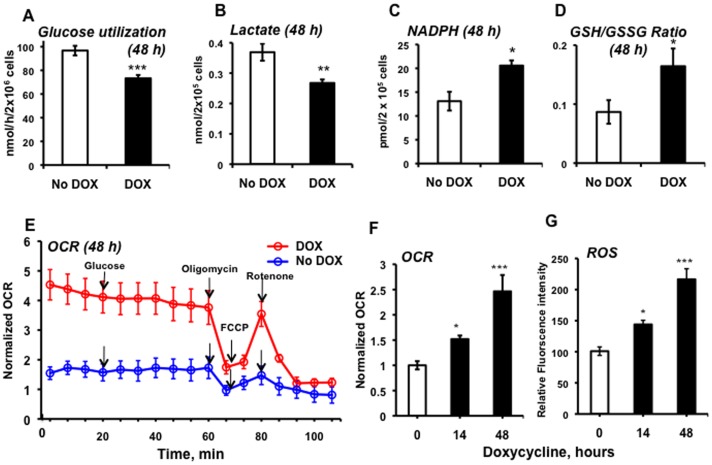
MTG16-expressing cells showed altered metabolism. Metabolism was assayed in Raji/MTG16 Tet-On 3G cells incubated without doxycycline (negative control) or 20 ng/ml doxycycline (to induce *MTG16* expression). **A**. Glycolytic metabolism was assessed by measuring glucose utilization, determined from the rate of [^3^H]OH production from [5-^3^H]glucose, and **B**. lactate production. **C**. The redox status of the cell was assessed by measuring NADPH production and **D**. the GSH/GSSG-ratio. **E-F**. Mitochondrial metabolism was assessed by measuring the oxygen consumption rate (OCR). Data are shown for cells incubated without doxycycline (blue) or cells incubated with 20 ng/ml doxycycline (red) for 48 h. The initial glucose concentration was 11.1 mmol/L and additional 11.1 mmol/L glucose was added as indicated with arrow. Oligomycin, an inhibitor of ATP synthase, was added to discriminate between ATP-linked respiration and proton leak. Subsequently, the maximal respiratory capacity was determined after adding of 4 µM FCCP, which is an uncoupler of mitochondrial oxidative phosphorylation that raises OCR to a maximal rate. Finally, 1 µM rotenone was added to block transfer of electrons from complex I to ubiquinone. **G**. Timing of ROS production. Data are represented as means±SEM for (A) n = 4, (B) n = 4, (C) n = 4, (D) n = 4, (E) n = 4, (F) n = 4, and (G) n = 4 and were compared by the unpaired Student's *t* test (*p<0.05; ***p<0.001).

To investigate the impact of MTG16 on glucose metabolism, we first measured glucose utilization as the rate of [^3^H]OH production from [5-^3^H] glucose. Indeed, glucose utilization was decreased in doxycycline–induced cells (Figure 6A). The decrease was consistent with the observed repression of *PFKFB3* with an expected diminished activation of PFK1. Furthermore, we found a decrease in lactate release into the extracellular medium (Figure 6B), further supporting a diminished glycolytic rate. The reduction in glycolytic activity caused by elevated MTG16 potentially explains the growth and cell cycle inhibition shown in Figure 1D,E.

MTG16-expression also increased the level of NADPH (Figure 6C), suggesting increased glucose flux through the PPP [54]. However, one can not rule out NADPH production by malic enzyme and isocitrate dehydrogenase. The increased level of NADPH was paralleled by an increased GSH/GSSG-ratio (Figure 6D), supporting the notion that the rise in NADPH levels alters the cellular redox state.

Next, we investigated mitochondrial metabolism, which under aerobic conditions is tightly coupled to glycolytic metabolism. To this end we employed The Seahorse Extracellular Flux Analyzer XF24 measuring the oxygen consumption rate (OCR), which reflects overall metabolic activity of mitochondria. Non-induced Raji/MTG16 Tet-On 3G cells showed a very low OCR (Figure 6E,F). This suggests that the Warburg effect, characteristic of cancer cells [20], prevailed in the cells. In contrast, cells expressing MTG16 showed increased OCR already at 14 h of incubation with doxycycline (Figure 6E,F). Both doxycycline induced- and non-induced cells showed a drop in OCR upon the injection of oligomycin. This drop was more pronounced in doxycycline-induced cells, suggesting a higher rate of mitochondrial ATP synthesis (and hence ATP turnover) in these cells. MTG16 expression did not further increase respiration in response to FCCP. This may be explained by a limitation of substrate availability for oxidative phosphorylation in these cells (Figure 6E). Addition of rotenone, inhibitor of the electron transfer from complex I to ubiquinone, showed that non-mitochondrial oxygen consumption is similar in doxycycline-induced and non-induced cells (Figure S3). Non-mitochondrial respiration may be caused by non-mitochondrial NADP oxidases and other enzymes such as desaturases and detoxification enzymes.

ROS levels began to increase after 14 h of *MTG16* expression, with a further increase with time (Figure 6G). Thus, ROS levels increased concomitantly with stimulation of mitochondrial respiration. Importantly, our results show that increased ROS production is paralleled by increases in NADPH levels and an elevated GSH/GSSG ratio, reflecting an expected cellular response to increased oxidative metabolism.

### Metabolite profiling

**Figure 7:**
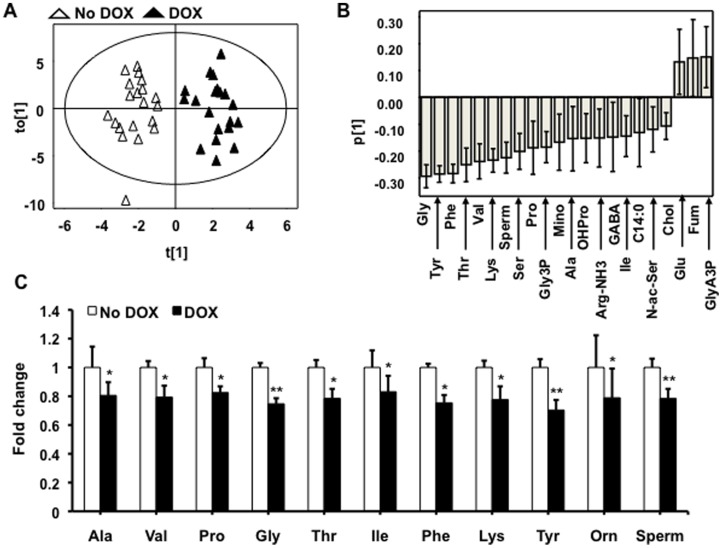
Metabolite profiling of MTG16-expressing cells. **A**. Metabolite data were analyzed by orthogonal projections to latent structures discriminant analysis (OPLS-DA). This model described 89% (R^2^(Y) = 0.89) of the variation in the sample class with a predictive ability of 84% (Q^2^(Y) = 0.84%). The OPLS-DA score scatter-plot revealed a perfect clustering of samples according to cell type. Thus, the metabolite pattern differed systematically between doxycycline non-induced (no DOX) and doxycycline–induced (DOX) cells. The position of each sample in this plot is determined by levels of all 61 detected metabolite derivatives. The axes t[1] and to[1] depict the first predictive and the first orthogonal component, respectively. Hence, differences in the metabolite pattern underlie the separation of groups along t[1], whereas separation along to[1] is due to other structured variation in the metabolite pattern not related to class belonging. **B**. The loading plot for the predictive variation (p[1]), describing which metabolites that differ in level between non-induced and induced cells, reveals that mainly amino acid levels are decreased in doxycycline induced cells. **C**. Raw data plots of metabolite levels normalized to protein and expressed as fold to control cells±SEM for n = 5. Statistical significance was assessed by the paired Student's *t*-test (*p<0.05, **p<0.01).

Metabolite profiling by GC/MS yielded data on 61 metabolite derivatives, corresponding to 55 unique metabolites. To generate an overview of the data, samples were classified by OPLS-DA according to their exposure to doxycycline. Clearly, samples could be accurately classified based on the metabolite pattern (Score scatter plot, Figure 7A), suggesting clear differences in the metabolite profiles between the control (no doxycycline) and the doxycycline–induced cells. The loading plot (Figure 7B) reveals changes in metabolite levels underlying the clustering of samples observed in the score scatter plot. Hence, this analysis showed a systematic decrease in levels of amino acids at 48 h of doxycycline induction. Significant down-regulation of amino acids by *MTG16* expression was confirmed by plotting relative levels of metabolites (Figure 7C). Thus, amino acid levels were decreased by on average 22% at 48 h. No decrease in levels of amino acids was observed at 24 h (data not shown), indicating that this effect is not an early response to MTG16.

### MTG16 increased phosphorylation of JNK1, ERK1/2 and p38 MAPKs

**Figure 8:**
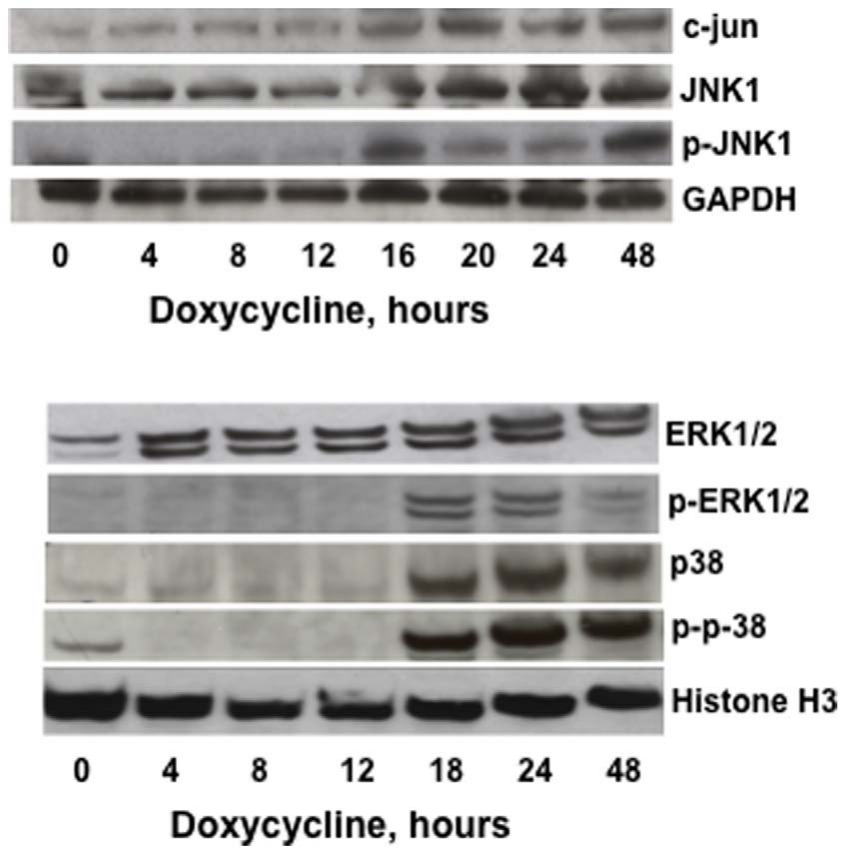
Activation of MAPKs in MTG16-expressing cells. The Raji/MTG16 Tet-On 3G cells were incubated with 20 ng/ml doxycycline up to 48 h. The content of c-Jun, JNK1, phosphorylated JNK1 (p-JNK), GAPDH, ERK1/2, phosphorylated ERK1/2 (p-ERK1/2), p38, phosphorylated p38 (p-p38) and Histone H3 was analyzed by Western blotting. The experiments were repeated at least three times and representative results are shown.

MAPKs are essential in the mediation of a variety of cellular responses such as metabolic and oxidative stress [55]. To investigate whether this occurs in our system, we assayed levels of MAPKs and their phosphorylated activated forms as a function of time by Western blotting after induction of MTG16 by doxycycline (Figure 8). The activated phosphorylated c-Jun amino–terminal kinase1 (p-JNK1), the major isoform responsible for c-Jun N-terminal phosphorylation [56], was increased after 18 to 24 h of incubation with doxycycline. This paralleled upregulation of c-Jun. The basal level of extracellular signal–regulated kinases (ERK1/2) was not affected. However, phosphorylated ERK1/2 (p-ERK1/2) increased after 18 to 24 h of doxycyclin incubation. The basal level of both p38 and phosphorylated p38 (p-p38) increased between 18 to 24 h of incubation with doxycycline and persisted for at least 48 h. This finding is consistent with the lack of upregulation of *p38* expression in the cDNA array, which was performed at 8 h of doxycycline incubation. Possibly, JNK1, p38 and ERK1/2 were activated upon MTG16 expression as an effect of stress secondary to the increased ROS production (Figure 6G) that preceded activation of MAPKs.

## Discussion

It is widely held that tumor cells exhibit altered metabolic activity, which facilitates uptake and incorporation of nutrients into macromolecules needed for cell division [21]. The increased nutrient uptake is balanced by increased lactate production to eliminate excess carbon and by reduced mitochondrial activity through inactivation of pyruvate dehydrogenase to avoid excess ROS production. By affecting critical metabolic control points, MTG16 was found to change metabolism towards that of non–transformed cells by down regulating glycolysis and augmenting mitochondrial respiration. Hence, it is possible that elevated expression of MTG16 may revert a Warburg–like effect. Conversely, lack of MTG16 activity through allele disruption [2], [3], [5], [6] or mutation [47], [48] could favour a Warburg–like effect in transformed cells. If inactivation of ETO homologues contributes to malignancy, restoration of their functions may hinder malignancy by promoting growth inhibition. Indeed, MTG16 seemed to produce a less malignant phenotype in B-lymphoblastoid Raji cells by reducing cell cycling and proliferation.

Protein expression of MTG16 was observed within 3–4 h of doxycycline incubation and a significant change of expression of genes was noted within 4 h (Figure S1). Our observations that expression levels of MTG16 protein were within physiological ranges, repression of genes was efficient and rapid, certain known genes to be directly regulated by MTG16 (*HES1*, *BCL6*) were downregulated, and specific Nervy Homology Regions of MTG16 were required intact for repression, may altogether suggest that the currently reported effects on metabolic regulators could be direct effects of MTG16. As MTG16 being a co-repressor is assumed to mediate repression by binding DNA binding transcription factors and recruiting its chromatin silencing machinery. Therefore, it is possible that MTG16 co-repressor binds to those transcription factors that are responsible for expression of the genes in question and then execute its co-repressor effect. However, it should be emphasized that we cannot exclude that the observed effects from MTG16 expression on transcription may involve indirect effects, such as squelching of critical transcription factors away from promoters.

Members of the ETO gene family share four evolutionary highly conserved Nervy Homology Regions (NHR 1 to 4) with distinctive attributes [57]. The NHR1 domain is a structural platform for interactions with positive and negative regulators of gene expression [58], the NHR2 domain is required for oligomerization with other ETO homologue proteins and for protein interaction with the transcriptional corepressor sin3A [57], the NHR3 domain may cooperate with other NHRs in co-repressor interactions [59] and the NHR4 domain binds HDACs and corepressor proteins such as N-CoR and SMRT [60], [61]. Diminished expression of genes for glycolytic stimulators required intact NHR2-3 of MTG16, indicating that protein–protein interactions with co-repressors and HDACs were required. Furthermore, NHR2-dependent oligomerization may increase the number of co-repressors recruited to the complex and strengthen transcriptional repression [62]. Inhibition of genes for glycolytic regulators did not require intact NHR1 and NHR4, thus indicating that the NHR1-mediated interactions with E-proteins [63] and the NHR4-specific interactions with nuclear co-repressors [60], [61] were not required. These results parallel previous findings [18], which by use of deletion mutants also mapped the major transcriptional repression activity of MTG16 to the central region of the protein including NHR2 and NHR3. Given 98% homology within NHR domains, a similar mapping data for repression might be expected in MTG16 and ETO. Indeed, results from transient transfections [59] showed that ETO required an intact core repressor domain, including NHR2 and NHR3, for maximal repressor activity; this is similar to our findings on MTG16. Additionally, the data in [59] suggested that multiple ETO regions may work in combination to repress transcription as the NHR regions lacked repression activity on their own.

The balance between the bifunctional PFKFB3 [50] and PFKFB4 [64], [65], [66] activities is critical to balance glycolytic and PPP-mediated glucose metabolism (Figure 5). PFKFB3 has a strong net kinase activity [66]; the observed repression of *PFKFB3* should result in reduced glycolysis due to lowered F-2,6-bisP production. The latter compound is an activator of PFK1, which is the key regulator in the mammalian glycolytic pathway (Figure 5) [67]. Thus, inhibition of PFKB3 expression may result in diminished allosteric activation of PFK1, reduced glycolytic consumption of G-6-P and a reduced glycolytic flux. Indeed, the inhibition of glycolysis by doxycycline–induced MTG16 was reflected by decreased glucose utilization and decreased lactate production. Decreased PFKFB4 levels, resulting in a lowered bisphosphatase activity, might predict a lower glucose flux in the PPP. However, NADPH and glutathione levels were increased by *MTG16* expression, suggesting that the PPP was intact. Inhibition of PFKFB3 with its strong net kinase activity would give less production of F-2,6-bisP from F-6-P and thereby support possible intact glucose flux through the PPP. Furthermore, the kinase to bisphosphatase ratio of PFKFB4 may vary and so the influence on the PPP [51], [52]. Additionally, NADPH might be produced by malic enzyme and the cytosolic form of isocitrate dehydrogenase.

Metabolic profiling revealed a shift of metabolism in MTG16 expressing cells. The observed systemic reduction in levels of amino acids was a delayed event of MTG16 expression. However, reduced availability of amino acids may contribute to the observed inhibition of proliferation.

The diminished expression of *PDK1* by MTG16 may explain enhanced mitochondrial respiration, which is a reflection of overall increased mitochondrial metabolism. PDK1 inhibits PDH (Figure 5), thereby reducing respiration [53]. Inhibition of *PDK1* expression would increase acetyl-CoA production, resulting in an increased activity in the tricarboxylic acid cycle. This would generate NADH, which drives the respiratory chain, indicated by increased consumption of O_2_. Thus, production of lactate would not be needed to reduce pyruvate and regenerate NAD^+^ to enable continued glycolysis. In fact, we silenced PDK1 in clonal insulin–producing insulinoma cells, which lead to enhanced TCA cycle activity and insulin secretion [68].

Decreased oxidative metabolism in tumor cells may explain a lack of production of superfluous ROS [69]. Hence, inhibition of *PDK1* expression by MTG16, leading to increased oxidative metabolism, may have contributed to the additional generation of ROS observed here. MTG16-induced metabolic changes, such as increased mitochondrial respiration and increased ROS production, were noted within 14 h of induction. In contrast, a retardation of the cell cycle was visible later, between 24 to 36 h of MTG16 induction. This sequence of events suggests that the preceding metabolic changes potentially underlie growth inhibition.

Activation of the JNK1, p38 and ERK1/2 MAPKs by phosphorylation as well as upregulation of JNK1 and p38 was initiated after 18 to 24 h of *MTG16* expression. This suggests that these changes may be indirect effects of MTG16 expression. The result is consistent with the lacking increase in *p38* expression at 8 h of incubation with doxycycline. The p38 MAPK plays a critical role in adaptation to oxidative stress by regulation of metabolism and control of gene expression eventually leading to cell cycle arrest and apoptosis [70]. Thus, our results suggest that JNK1, ERK1/2 and p38 were activated upon *MTG16* expression as a stress response secondary to a preceding increase of ROS (Figure 6G).

It is intriguing that elevation of MTG16 promoted a decrease in overall metabolism. Importantly, our protein expression system did not have cytotoxic effects; cell viability was not affected and apoptosis was not detected. Furthermore, levels of *MTG16* transcripts were similar in doxycycline–induced Raji cells and normal CD34-positive hematopoietic progenitor cells or erythroid and megakaryocytic cell lines. It is compelling that elevated *MTG16* expression also causes reduced ribogenesis [71] as increased ribosomal RNA (rRNA) synthesis/ribogenesis is a hallmark of cancer. The counteraction of MYC-driven activation of rRNA transcription pointed to a possible tumor suppressor function of MTG16. In short, observations made in different cell types indicate that MTG16 can affect cell metabolism at multiple levels.

In conclusion, we showed that the transcriptional nuclear co-repressor MTG16 diminished glycolysis and enhanced mitochondrial respiration. The increased flow of O_2_ in the respiratory chain coincided with increased ROS formation. The phosphorylation of the p38, JNK1 and ERK1/2 MAPKs was increased as a possible response to the metabolic and oxidative changes. Our results suggest that MTG16, by affecting the expression of several key glycolytic regulator genes, directly or indirectly, may serve as a brake on glycolysis, an enhancer of mitochondrial respiration and inhibitor of cell proliferation. Reconstitution or elevation of MTG16 and other ETO homologues might revert the Warburg effect by downregulation of PDK1 expression in transformed cells. This may exert an anti–tumor effect.

## Supporting Information

Figure S1 **Time course for expression of genes in Raji/MTG16 Tet-On 3G and Raji/Tet-3G control cells. A**. RT-qPCR was performed using RNA from Raji/MTG16 Tet-On 3G cells incubated 0, 4, 8 and 24 h with 20 ng/ml doxycycline for induction of MTG16. GAPDH, 18 S and ß-actin were used as housekeeping genes and relative mRNA expression was calculated by the ΔΔCT method taking 0 h uninduced cells as control. A number of genes investigated were downregulated by doxycyclin-induced MTG16 expression, PKM2 was upregulated. Data are represented as means±SEM for n = 3 and compared by the one-way ANOVA followed by the Dunnett's post-hoc test (*p<0.05; ***p<0.001). **B**. RT-qPCR was performed using RNA from Raji/Tet-3G control cells incubated for 24 hours with and without 20 ng/ml doxycycline (Dox). Expression of the examined genes was unaffected upon incubation with doxycycline. Thus, inhibited gene expression shown in A is associated with elevated MTG16 expression and not a Tet-transactivator effect. Data are represented as means±SEM for n = 3. **C**. Raji/MTG16 Tet-On 3G cells were incubated with 20 ng/ml doxycycline during 6 days and examined for viability and apoptosis. Cell viability was examined by flow cytometry using 7-amino actinomycin D as a label for dead cells. Apoptosis was examined by flow cytometry using fluorescently labelled annexin-V and 4′,6-diamidino-2-phenylindole (DAPI) staining as a label of apoptotic cells. Cell viability and apoptosis was unaffected during doxycycline-induced *MTG16* expression. Data are represented as means±SEM for n = 5.
(TIF)

Figure S2 ***MTG16*** **mRNA expression in various hematopoietic cell lines and CD34+ hematopoietic progenitor cells compared to Raji/MTG16 Tet-On 3G cells.** Data are represented as means±SEM for n = 3.
(TIF)

Figure S3 **Mitochondrial coupling and respiratory control in MTG16-expressing Raji/MTG16 Tet-On 3G cells.** The cells were incubated without doxycycline (DOX) (negative control) or 20 ng/ml doxycycline (to induce *MTG16* expression) for 48 h. Basal respiration, ATP turnover, proton leak, coupling efficiency, maximum respiration rate, apparent respiratory control ratio and non-mitochondrial respiration were determined by sequential addition of oligomycin (ATP synthase inhibitor), FCCP (uncoupler) and rotenone (electron transport inhibitor). Basal respiration (A), maximum respiratory rate (C) and ATP turnover (oligomycin–sensitive respiration) (D) were significantly increased in MTG16 expressing cells. No difference was observed in non–mitochondrial respiration (B), coupling efficiency (E) and proton leak (E) between control cells and cells expressing MTG16. Data are given as fold change represented as means±SEM for A to F (n = 4) and compared by the paired Student's *t* test (*p<0.05; ***p<0.001).
(TIF)

Table S1 Primers used for RT-PCR. Primers were designed using Primers 3 from exon-intron boundaries avoiding amplification of genomic DNA.
(DOC)

Table S2 Gene expression changes upon MTG16 expression in Raji/MTG16 Tet-On 3G cells. The list shows genes that were downregulated at least 0.8-fold or upregulated at least 1.3-fold with the average fold change between three biological replicate microarrays depicted as a ratio to 1.
(DOC)
