# Supplementary figures and images for: The Transcriptional Co-Repressor Myeloid Translocation Gene 16 Inhibits Glycolysis and Stimulates Mitochondrial Respiration
Source: PLoS One. 2013 Jul 1;8(7):e68502. doi: 10.1371/journal.pone.0068502 (PMC3698176; doi:10.1371/journal.pone.0068502)

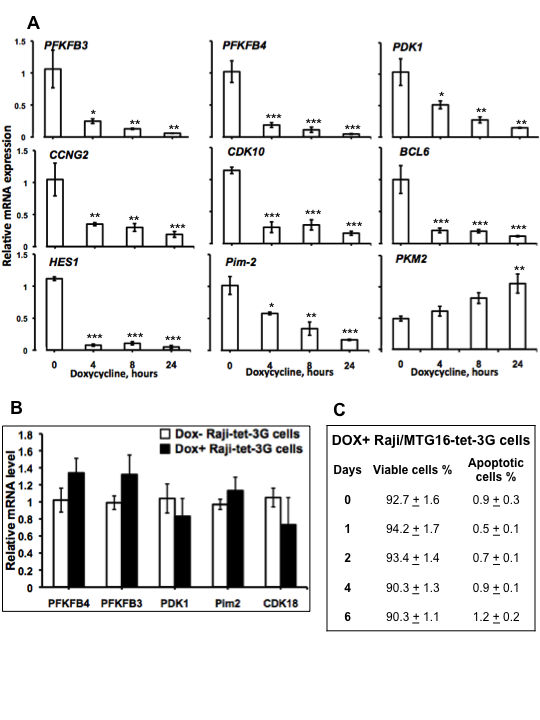

Supplement: Figure S1 — Time course for expression of genes in Raji/MTG16 Tet-On 3G and Raji/Tet-3G control cells. A. RT-qPCR was performed using RNA from Raji/MTG16 Tet-On 3G cells incubated 0, 4, 8 and 24 h with 20 ng/ml doxycycline for induction of MTG16. GAPDH, 18 S and ß-actin were used as housekeeping genes and relative mRNA expression was calculated by the ΔΔCT method taking 0 h uninduced cells as control. A number of genes investigated were downregulated by doxycyclin-induced MTG16 expression, PKM2 was upregulated. Data are represented as means±SEM for n = 3 and compared by the one-way ANOVA followed by the Dunnett's post-hoc test (*p<0.05; ***p<0.001). B. RT-qPCR was performed using RNA from Raji/Tet-3G control cells incubated for 24 hours with and without 20 ng/ml doxycycline (Dox). Expression of the examined genes was unaffected upon incubation with doxycycline. Thus, inhibited gene expression shown in A is associated with elevated MTG16 expression and not a Tet-transactivator effect. Data are represented as means±SEM for n = 3. C. Raji/MTG16 Tet-On 3G cells were incubated with 20 ng/ml doxycycline during 6 days and examined for viability and apoptosis. Cell viability was examined by flow cytometry using 7-amino actinomycin D as a label for dead cells. Apoptosis was examined by flow cytometry using fluorescently labelled annexin-V and 4′,6-diamidino-2-phenylindole (DAPI) staining as a label of apoptotic cells. Cell viability and apoptosis was unaffected during doxycycline-induced MTG16 expression. Data are represented as means±SEM for n = 5. (TIF) [file pone.0068502.s001.tif]
