# Supplementary material for: The Transcriptional Co-Repressor Myeloid Translocation Gene 16 Inhibits Glycolysis and Stimulates Mitochondrial Respiration
Source: PLoS One. 2013 Jul 1;8(7):e68502. doi: 10.1371/journal.pone.0068502 (PMC3698176; doi:10.1371/journal.pone.0068502)
Supplement: Table S1 — Primers used for RT-PCR. Primers were designed using Primers 3 from exon-intron boundaries avoiding amplification of genomic DNA. (DOC) [file pone.0068502.s004.doc]

**Table S1**

| **Probe Set ID** | **UniGene ID** | **Gene Description** | **Gene Symbol** | **mRNA Accession** | **Fold Change** | | **q-value (%)** | **P-value** |
| --- | --- | --- | --- | --- | --- | --- | --- | --- |
| **Downregulated Genes** | | | | | | | | |
| 8123976 | Hs.484686 | t-complex 11 (mouse)-like 2 | TCP11L2 | NM_152772 | | 0.14 | 0 | 0.015 |
| 8150881 | Hs.14968 | jumonji C domain containing histone demethylase 1 homolog D (S. cerevisiae) | JHDM1D | NM_030647 | | 0.17 | 0 | 4.31E-04 |
| 7918487 | Hs.557850 | 6-phosphofructo-2-kinase/fructose-2,6-biphosphatase 4 | PFKFB4 | NM_004567 | | 0.18 | 0 | 0.008 |
| 7928308 | Hs.712599 | zinc finger CCCH-type containing 6 | ZC3H6 | NM_198581 | | 0.21 | 0 | 0.002 |
| 8133233 | Hs.21631 | DNA-damage-inducible transcript 4 | DDIT4 | NM_019058 | | 0.22 | 0 | 0.005 |
| 8071907 | Hs.474388 | chromosome 7 open reading frame 68 | C7orf68 | NM_013332 | | 0.25 | 0 | 0.004 |
| 8173217 | Hs.54697 | kelch-like 6 (Drosophila) | KLHL6 | NM_130446 | | 0.28 | 0 | 0.003 |
| 8137693 | Hs.121593 | guanine nucleotide binding protein (G protein), gamma 7 | GNG7 | NM_052847 | | 0.29 | 0 | 0.017 |
| 8133518 | Hs.647047 | KIAA1147 | KIAA1147 | NM_001080392 | | 0.29 | 0 | 4.62E-04 |
| 8107133 | Hs.369430 | WD repeat and SOCS box-containing 1 | WSB1 | NM_015626 | | 0.31 | 0 | 0.0028 |
| 8164810 | Hs.106185 | hairy and enhancer of split 1, (Drosophila) | HES1 | NM_005524 | | 0.31 | 0 | 0.0181 |
| 7943376 | Hs.156352 | pim-2 oncogene | PIM2 | NM_006875 | | 0.33 | 0 | 0.0239 |
| 8073909 | Hs.435044 | T-cell activation RhoGTPase activating protein | TAGAP | NM_054114 | | 0.33 | 0 | 0.0103 |
| 8172471 | Hs.496096 | NUAK family, SNF1-like kinase, 2 | NUAK2 | NM_030952 | | 0.33 | 0 | 0.0037 |
| 7963721 | Hs.156667 | myosin regulatory light chain interacting protein | MYLIP | NM_013262 | | 0.35 | 0 | 0.0015 |
| 8087485 | Hs.16695 | ephrin-A3 | EFNA3 | NM_004952 | | 0.35 | 0 | 0.0062 |
| 8080445 | Hs.420257 | deoxyribonuclease I-like 3 | DNASE1L3 | NM_004944 | | 0.36 | 0 | 0.0020 |
| 7987454 | Hs.591104 | myosin IG | MYO1G | NM_033054 | | 0.37 | 0 | 0.0023 |
| 8144625 | Hs.146591 | zinc finger and BTB domain containing 20 | ZBTB20 | NM_015642 | | 0.37 | 0 | 0.0017 |
| 7947624 | Hs.502458 | leukocyte immunoglobulin-like receptor, subfamily B (with TM and ITIM domains), member 1 | LILRB1 | NM_006669 | | 0.38 | 0 | 0.0050 |
| 7999423 | Hs.50640 | lysine (K)-specific demethylase 3A | KDM3A | NM_018433 | | 0.38 | 0 | 0.0017 |
| 8010903 | Hs.651925 | dual specificity phosphatase 22 | DUSP22 | NM_020185 | | 0.39 | 0 | 0.0230 |
| 8015798 | Hs.546897 // Hs.640133 | B-cell CLL/lymphoma 6 | BCL6 | NM_001706 | | 0.39 | 0 | 0.0011 |
| 7905918 | Hs.516656 | leukocyte-associated immunoglobulin-like receptor 1 | LAIR1 | NM_002287 | | 0.40 | 0 | 0.0027 |
| 8171087 | Hs.124942 | 6-phosphofructo-2-kinase/fructose-2,6-biphosphatase 3 | PFKFB3 | NM_004566 | | 0.40 | 0 | 0.0041 |
| 8016745 | Hs.463439 | fragile histidine triad gene | FHIT | NM_002012 | | 0.40 | 0 | 0.0215 |
| 8117900 | Hs.631988 | yippee-like 3 (Drosophila) | YPEL3 | NM_031477 | | 0.40 | 0 | 0.008 |
| 7928019 | Hs.370365 | pyruvate dehydrogenase kinase, isozyme 1 | PDK1 | NM_002610 | | 0.41 | 0 | 0.0017 |
| 8089659 | Hs.477159 | adenosine deaminase | ADA | NM_000022 | | 0.41 | 0 | 0.0108 |
| 7908917 | Hs.519162 | taxilin beta | TXLNB | NM_153235 | | 0.42 | 0 | 0.0068 |
| 8143341 | Hs.308710 | membrane-associated ring finger (C3HC4) 1 | 1-Mar | NM_017923 | | 0.43 | 0 | 1.61E-04 |
| 8092691 | Hs.478588 | huntingtin interacting protein 1 | HIP1 | NM_005338 | | 0.43 | 0 | 4.85E-04 |
| 8037563 | Hs.466937 | neurexin 3 | NRXN3 | NM_004796 | | 0.43 | 0 | 0.0025 |
| 8038326 | Hs.130949 | pellino homolog 1 (Drosophila) | PELI1 | NM_020651 | | 0.43 | 0 | 0.0209 |
| 8113157 | Hs.482868 | adducin 3 (gamma) | ADD3 | NM_016824 | | 0.43 | 0 | 0.0256 |
| 8115543 | Hs.573143 | tetratricopeptide repeat domain 21A | TTC21A | NM_145755 | | 0.43 | 0 | 0.0232 |
| 8119435 | Hs.194721 | SATB homeobox 1 | SATB1 | NM_002971 | | 0.43 | 0 | 0.0159 |
| 8122933 | Hs.586279 | cyclin-dependent kinase 18 | CDK18 | NM_212503 | | 0.44 | 0 | 0.0094 |
| 8113504 | Hs.36053 // Hs.483067 // Hs.694860 | cyclin G2 | CCNG2 | NM_004354 | | 0.44 | 0 | 0.0057 |
| 7930380 | Hs.501012 | citrate lyase beta like | CLYBL | NM_206808 | | 0.44 | 0 | 0.0051 |
| 8056060 | Hs.470369 | neutrophil cytosolic factor 1 | NCF1 | NM_000265 | | 0.44 | 0 | 0.0064 |
| 8145454 | Hs.131226 | neutrophil cytosolic factor 1 | NCF1 | NM_000265 | | 0.44 | 0 | 0.0064 |
| 7984846 | Hs.584748 | inhibitor of DNA binding 2, dominant negative helix-loop-helix protein | ID2 | NM_002166 | | 0.44 | 0 | 0.0065 |
| 8164607 | Hs.189409 | neutrophil cytosolic factor 1 | NCF1 | NM_000265 | | 0.45 | 0 | 0.0054 |
| 8130539 | Hs.529984 | pleiomorphic adenoma gene 1 | PLAG1 | NM_002655 | | 0.45 | 0 | 8.20E-04 |
| 7924549 | Hs.473317 | glucocorticoid induced transcript 1 | GLCCI1 | NM_138426 | | 0.45 | 0 | 5.04E-04 |
| 8064261 | Hs.2859 | Gardner-Rasheed feline sarcoma viral (v-fgr) oncogene homolog | FGR | NM_005248 | | 0.45 | 0 | 4.14E-04 |
| 7981745 | Hs.375094 | ubiquitin specific peptidase 28 | USP28 | NM_020886 | | 0.46 | 0 | 0.0124 |
| 8044375 | Hs.469658 | zinc finger protein 292 | ZNF292 | NM_015021 | | 0.47 | 0 | 0.0132 |
| 8069057 | Hs.255093 | PHD finger protein 21A | PHF21A | NM_001101802 | | 0.47 | 0 | 0.0244 |
| 8127364 | Hs.561539 | germinal center expressed transcript 2 | GCET2 | NM_152785 | | 0.47 | 0 | 0.0041 |
| 8108099 | Hs.595540 | phosphoinositide-3-kinase interacting protein 1 | PIK3IP1 | NM_052880 | | 0.47 | 0 | 0.0167 |
| 7995631 | Hs.513609 | interferon-induced protein 44-like | IFI44L | NM_006820 | | 0.47 | 0 | 0.0022 |
| 8111698 | Hs.407926 | zinc finger protein 608 | ZNF608 | NM_020747 | | 0.47 | 0 | 0.0026 |
| 8077036 | Hs.530380 | chromosome 19 open reading frame 54 | C19orf54 | NM_198476 | | 0.48 | 0 | 0.0044 |
| 7928395 | Hs.588854 | hypothetical LOC100130581 | LOC100130581 | NR_027413 | | 0.48 | 0 | 0.0019 |
| 8112220 | Hs.117545 // Hs.721404 | regulator of G-protein signaling 9 | RGS9 | NM_003835 | | 0.48 | 0 | 0.0051 |
| 8020323 | Hs.592347 | macrophage stimulating 1 (hepatocyte growth factor-like) | MST1 | NM_020998 | | 0.49 | 0 | 0.0165 |
| 8122660 | Hs.657370 | fucosyltransferase 11 (alpha (1,3) fucosyltransferase) | FUT11 | NM_173540 | | 0.49 | 0 | 0.0129 |
| 8101587 | Hs.125503 | family with sequence similarity 162, member A | FAM162A | NM_014367 | | 0.49 | 0 | 0.0046 |
| 7997427 | Hs.724370 | sodium channel, voltage-gated, type IV, alpha subunit | SCN4A | NM_000334 | | 0.49 | 0 | 0.0070 |
| 7936115 | Hs.602894 | proline-rich nuclear receptor coactivator 1 | PNRC1 | NM_006813 | | 0.49 | 0 | 0.0058 |
| 8131996 | Hs.437075 | transmembrane protein 120A | TMEM120A | NM_031925 | | 0.50 | 0 | 0.0085 |
| 8009277 | Hs.664380 | septin 3 | 3-Sep | NM_019106 | | 0.50 | 0 | 0.0029 |
| 7971780 | Hs.409989 | R3H domain containing 2 | R3HDM2 | NM_014925 | | 0.50 | 0 | 0.0106 |
| 8073548 | Hs.120483 | nuclear receptor subfamily 4, group A, member 1 | NR4A1 | NM_002135 | | 0.51 | 0 | 0.0018 |
| 8043491 | Hs.631517 | lysine (K)-specific demethylase 4B | KDM4B | NM_015015 | | 0.51 | 0 | 0.0059 |
| 8064868 | Hs.636359 | polycomb group ring finger 3 | PCGF3 | NM_006315 | | 0.51 | 0 | 0.0134 |
| 8085716 | Hs.517717 | solute carrier family 2 (facilitated glucose transporter), member 3 | SLC2A3 | NM_006931 | | 0.52 | 0 | 0.0023 |
| 7987405 | Hs.591127 | Bcl2 modifying factor | BMF | NM_001003940 | | 0.52 | 0 | 0.0132 |
| 7969815 | Hs.655642 | suppressor of cytokine signaling 1 | SOCS1 | NM_003745 | | 0.52 | 0 | 0.0026 |
| 7958262 | Hs.696047 | rearranged L-myc fusion | RLF | NM_012421 | | 0.52 | 0 | 9.62E-04 |
| 8031213 | Hs.710507 | ras homolog gene family, member H | RHOH | NM_004310 | | 0.52 | 0 | 0.0173 |
| 7932132 | Hs.330463 // Hs.656573 | glucuronidase, beta pseudogene 4 | GUSBP4 | NR_003660 | | 0.52 | 0 | 0.0052 |
| 8176986 | Hs.124942 | peptidylglycine alpha-amidating monooxygenase | PAM | NM_000919 | | 0.53 | 0 | 0.0042 |
| 7999496 | Hs.371856 | polo-like kinase 1 substrate 1 | PLK1S1 | NM_018474 | | 0.53 | 0 | 0.0188 |
| 7972003 | Hs.373857 | ureidopropionase, beta | UPB1 | NM_016327 | | 0.54 | 0 | 0.0173 |
| 8113491 | Hs.93842 | transmembrane protein 8B | TMEM8B | NM_016446 | | 0.54 | 0 | 0.0018 |
| 7976012 | Hs.368307 | family with sequence similarity 65, member B | FAM65B | NM_014722 | | 0.54 | 0 | 0.0014 |
| 8078857 | Hs.443935 | zinc finger and BTB domain containing 25 | ZBTB25 | NM_006977 | | 0.55 | 0 | 0.0045 |
| 7911347 | Hs.453629 | tetratricopeptide repeat, ankyrin repeat and coiled-coil containing 1 | TANC1 | NM_033394 | | 0.55 | 0 | 0.0031 |
| 7974621 | Hs.161000 | opiate receptor-like 1 | OPRL1 | NM_182647 | | 0.55 | 0 | 4.09E-04 |
| 8084880 | Hs.250666 | myelin basic protein | MBP | NM_001025101 | | 0.56 | 0 | 0.0022 |
| 8110670 | Hs.453629 | RPTOR independent companion of MTOR, complex 2 | RICTOR | NM_152756 | | 0.56 | 0 | 0.0031 |
| 8093112 | Hs.518524 | hexokinase 1 | HK1 | NM_033500 | | 0.57 | 0 | 0.0093 |
| 8040103 | Hs.180919 | F-box protein 25 | FBXO25 | ENST00000276326 | | 0.57 | 0 | 0.0039 |
| 8113761 | Hs.266616 | calcium binding and coiled-coil domain 1 | CALCOCO1 | NM_020898 | | 0.58 | 0 | 0.0016 |
| 8026365 | Hs.515215 | family with sequence similarity 116, member B | FAM116B | NM_001001794 | | 0.58 | 0 | 0.0165 |
| 8031223 | Hs.667388 | protein-L-isoaspartate (D-aspartate) O-methyltransferase domain containing 2 | PCMTD2 | BC032332 | | 0.58 | 0 | 3.87E-04 |
| 8086961 | Hs.476217 | protein-L-isoaspartate (D-aspartate) O-methyltransferase domain containing 2 | PCMTD2 | BC032332 | | 0.58 | 0 | 3.87E-04 |
| 8075483 | Hs.26670 | protein tyrosine phosphatase, receptor type, M | PTPRM | NM_001105244 | | 0.59 | 0 | 0.0027 |
| 8028286 | Hs.655502 | prolyl 4-hydroxylase, alpha polypeptide II | P4HA2 | NM_004199 | | 0.59 | 0 | 0.0096 |
| 8020702 | Hs.369519 | diacylglycerol kinase, delta 130kDa | DGKD | NM_152879 | | 0.59 | 0 | 0.0092 |
| 8038126 | Hs.428446 | cytochrome P450, family 2, subfamily E, polypeptide 1 | CYP2E1 | NM_000773 | | 0.59 | 0 | 0.0034 |
| 8121076 | Hs.75969 | protein phosphatase 2, regulatory subunit B'', beta | PPP2R3B | NM_013239 | | 0.59 | 0 | 0.0041 |
| 8116559 | Hs.401013 | mannose phosphate isomerase | MPI | NM_002435 | | 0.59 | 0 | 0.0060 |
| 8131496 | Hs.131673 | AT rich interactive domain 4A (RBP1-like) | ARID4A | NM_002892 | | 0.59 | 0 | 2.49E-04 |
| 8143307 | Hs.724392 | mitogen-activated protein kinase 10 | MAPK10 | NM_138982 | | 0.59 | 0 | 0.0163 |
| 8029701 | Hs.532872 | septin 14 | 14-Sep | ENST00000388975 | | 0.59 | 0 | 0.0133 |
| 8030539 | Hs.631587 | septin 14 | 14-Sep | ENST00000388975 | | 0.60 | 0 | 0.0133 |
| 8061919 | Hs.153934 | septin 14 | 14-Sep | ENST00000388975 | | 0.60 | 0 | 0.0133 |
| 7968417 | Hs.507669 | septin 14 | 14-Sep | ENST00000388975 | | 0.60 | 0 | 0.0133 |
| 8154100 | Hs.370422 | septin 14 | 14-Sep | ENST00000388975 | | 0.60 | 0 | 0.0133 |
| 8013159 | Hs.462379 | septin 14 | 14-Sep | ENST00000388975 | | 0.60 | 0 | 0.0133 |
| 8087182 | Hs.655006 | septin 14 | 14-Sep | ENST00000388975 | | 0.60 | 0 | 0.0133 |
| 8039034 | Hs.655967 | septin 14 | 14-Sep | ENST00000388975 | | 0.60 | 0 | 0.0133 |
| 8088958 | Hs.436062 | protein tyrosine phosphatase type IVA, member 3 | PTP4A3 | NM_032611 | | 0.60 | 0 | 0.0158 |
| 7896742 | Hs.453629 | carbonic anhydrase XI | CA11 | NM_001217 | | 0.60 | 0 | 0.0033 |
| 8094743 | Hs.654594 | Cdc42 guanine nucleotide exchange factor (GEF) 9 | ARHGEF9 | NM_015185 | | 0.61 | 0 | 0.0035 |
| 7912166 | Hs.463041 | dedicator of cytokinesis 6 | DOCK6 | NM_020812 | | 0.61 | 0 | 0.0050 |
| 8148964 | Hs.453629 | non-protein coding RNA 266 | NCRNA00266 | BC118988 | | 0.61 | 0 | 0.0031 |
| 8134869 | Hs.202097 | G protein-coupled receptor kinase 5 | GRK5 | NM_005308 | | 0.61 | 0 | 0.0016 |
| 8145005 | Hs.106124 | B lymphoid tyrosine kinase | BLK | NM_001715 | | 0.61 | 0 | 0.0078 |
| 8084232 | Hs.632575 | protein phosphatase 2, regulatory subunit B'', beta | PPP2R3B | NM_013239 | | 0.61 | 0 | 0.0139 |
| 7993223 | Hs.35490 | phosphodiesterase 4D, cAMP-specific | PDE4D | NM_001165899 | | 0.62 | 0 | 0.0166 |
| 7902541 | Hs.724492 | inter-alpha (globulin) inhibitor H1 | ITIH1 | NM_002215 | | 0.62 | 0 | 0.0015 |
| 7984922 | Hs.75694 | StAR-related lipid transfer (START) domain containing 4 | STARD4 | NM_139164 | | 0.63 | 0 | 0.0014 |
| 8080144 | Hs.271742 | calcium channel, voltage-dependent, beta 4 subunit | CACNB4 | NM_000726 | | 0.63 | 0 | 0.0160 |
| 8086330 | Hs.370950 | septin 14 | 14-Sep | ENST00000388975 | | 0.63 | 0 | 0.0057 |
| 8097064 | --- | protein phosphatase 2, regulatory subunit B', beta | PPP2R5B | NM_006244 | | 0.64 | 0 | 6.12E-04 |
| 7931643 | Hs.12907 | stathmin 1 | STMN1 | NM_001145454 | | 0.64 | 0 | 0.0030 |
| 8093398 | Hs.144309 | SRY (sex determining region Y)-box 5 | SOX5 | NM_152989 | | 0.64 | 0 | 0.0023 |
| 8033912 | Hs.202672 | mediator complex subunit 26 | MED26 | NM_004831 | | 0.64 | 0 | 0.0024 |
| 8103508 | Hs.592804 | ubiquitin protein ligase E3 component n-recognin 5 | UBR5 | NM_015902 | | 0.65 | 0 | 2.51E-04 |
| 8143919 | Hs.647067 | sperm associated antigen 4 | SPAG4 | NM_003116 | | 0.65 | 0 | 0.0147 |
| 8085054 | Hs.453629 | slingshot homolog 2 (Drosophila) | SSH2 | NM_033389 | | 0.65 | 0 | 0.0032 |
| 8037970 | Hs.631562 | MAP kinase interacting serine/threonine kinase 2 | MKNK2 | NM_199054 | | 0.65 | 0 | 0.0074 |
| 8124280 | Hs.559459 | retinoblastoma-like 2 (p130) | RBL2 | NM_005611 | | 0.66 | 0 | 0.0011 |
| 8074780 | Hs.517436 | zinc finger, FYVE domain containing 20 | ZFYVE20 | NM_022340 | | 0.66 | 0 | 0.0088 |
| 8049317 | Hs.471675 | zinc finger protein 676 | ZNF676 | NM_001001411 | | 0.66 | 0 | 0.0021 |
| 7945377 | Hs.501624 | synaptotagmin-like 3 | SYTL3 | NM_001009991 | | 0.66 | 0 | 0.0111 |
| 8032392 | Hs.515032 | spastic paraplegia 11 (autosomal recessive) | SPG11 | NM_025137 | | 0.67 | 0 | 0.0023 |
| 8087925 | Hs.118845 | arginine-glutamic acid dipeptide (RE) repeats | RERE | NM_012102 | | 0.67 | 0 | 0.0137 |
| 7907058 | Hs.493649 | PR domain containing 2, with ZNF domain | PRDM2 | NM_012231 | | 0.67 | 0 | 0.0107 |
| 8042942 | Hs.406266 // Hs.591588 | protein phosphatase 1, regulatory (inhibitor) subunit 13 like | PPP1R13L | NM_006663 | | 0.67 | 0 | 0.0072 |
| 8179184 | Hs.631988 | chromosome 6 open reading frame 204 | C6orf204 | NM_001178035 | | 0.68 | 0 | 0.0097 |
| 8137666 | Hs.453629 | 5'-3' exoribonuclease 1 | XRN1 | NM_019001 | | 0.68 | 0 | 0.0030 |
| 8066431 | Hs.654536 | basonuclin 2 | BNC2 | NM_017637 | | 0.68 | 0 | 0.0010 |
| 8110032 | Hs.484195 | deltex homolog 1 (Drosophila) | DTX1 | NM_004416 | | 0.69 | 0 | 0.0126 |
| 8155148 | Hs.493808 | lysine (K)-specific demethylase 4C | KDM4C | NM_015061 | | 0.69 | 0 | 0.0015 |
| 8088458 | Hs.655995 | procollagen C-endopeptidase enhancer | PCOLCE | NM_002593 | | 0.69 | 0 | 0.0036 |
| 8135915 | Hs.706124 | inositol 1,4,5-trisphosphate 3-kinase B | ITPKB | NM_002221 | | 0.69 | 0 | 0.0015 |
| 8043283 | Hs.557425 | DNA (cytosine-5-)-methyltransferase 1 | DNMT1 | NM_001130823 | | 0.71 | 0 | 7.31E-04 |
| 7909104 | Hs.445402 | tau tubulin kinase 2 | TTBK2 | NM_173500 | | 0.74 | 0 | 9.15E-04 |
| 8152148 | Hs.492445 | hypothetical LOC401884 | MGC57346 | NR_026680 | | 0.75 | 0 | 6.51E-04 |
| **Upregulated Genes** | | | | | | | | |
| 8091141 | Hs.435103 | solute carrier family 25, member 13 (citrin) | SLC25A13 | NM_001160210 | | 1.29 | 0 | 0.0049 |
| 8133314 | Hs.647047 | zinc finger protein 780A | ZNF780A | NM_001142577 | | 1.30 | 0 | 5.58E-04 |
| 8089701 | Hs.655108 | leucine-rich repeats and WD repeat domain containing 1 | LRWD1 | NM_152892 | | 1.30 | 0 | 6.62E-04 |
| 8139433 | Hs.37617 | zinc finger protein 597 | ZNF597 | NM_152457 | | 1.35 | 0 | 8.40E-04 |
| 8140319 | Hs.329266 | mitochondrial translational release factor 1-like | MTRF1L | NM_019041 | | 1.35 | 0 | 2.79E-04 |
| 8132055 | Hs.200100 | zinc finger protein 7 | ZNF7 | NM_003416 | | 1.37 | 0 | 0.0091 |
| 8024909 | Hs.654816 | sedoheptulokinase | SHPK | NM_013276 | | 1.37 | 0 | 0.0043 |
| 8045889 | Hs.61590 | KIAA1967 | KIAA1967 | NM_021174 | | 1.40 | 0 | 4.02E-04 |
| 8117020 | Hs.484738 | WD repeat domain 81 | WDR81 | NM_001163809 | | 1.42 | 0 | 5.73E-04 |
| 8120992 | Hs.485892 // Hs.656621 | La ribonucleoprotein domain family, member 4 | LARP4 | NM_052879 | | 1.42 | 0 | 0.0018 |
| 8089478 | Hs.49614 | cyclin J | CCNJ | NM_001134375 | | 1.42 | 0 | 0.0024 |
| 8017521 | Hs.46038 | kelch-like 18 (Drosophila) | KLHL18 | NM_025010 | | 1.42 | 0 | 7.94E-04 |
| 8143441 | Hs.521240 | dipeptidyl-peptidase 9 | DPP9 | NM_139159 | | 1.43 | 0 | 2.60E-04 |
| 8160260 | Hs.656581 // Hs.693436 | spermidine synthase | SRM | NM_003132 | | 1.43 | 0 | 0.0023 |
| 7914112 | Hs.1422 | vacuolar protein sorting 25 homolog (S. cerevisiae) | VPS25 | NM_032353 | | 1.43 | 0 | 3.48E-04 |
| 8147101 | Hs.445758 | transforming growth factor, beta 1 | TGFB1 | NM_000660 | | 1.46 | 0 | 0.0105 |
| 8044450 | Hs.190477 | zinc finger protein 417 | ZNF417 | NM_152475 | | 1.47 | 0 | 4.41E-04 |
| 8132439 | Hs.586313 | zinc finger and BTB domain containing 9 | ZBTB9 | NM_152735 | | 1.49 | 0 | 0.0106 |
| 8035825 | Hs.55452 | CD164 molecule, sialomucin | CD164 | NM_006016 | | 1.49 | 0 | 0.0033 |
| 8056909 | Hs.592510 | importin 4 | IPO4 | NM_024658 | | 1.49 | 0 | 0.0126 |
| 8177867 | Hs.631988 | tetratricopeptide repeat domain 27 | TTC27 | NM_017735 | | 1.49 | 0 | 0.0116 |
| 8039257 | Hs.572535 | WD repeat domain 55 | WDR55 | NM_017706 | | 1.50 | 0 | 0.0014 |
| 7998542 | Hs.389438 | tripartite motif-containing 27 | TRIM27 | NM_006510 | | 1.50 | 0 | 0.0098 |
| 8093440 | Hs.193326 | tripartite motif-containing 27 | TRIM27 | NM_006510 | | 1.50 | 0 | 0.0098 |
| 8092392 | Hs.659147 | polymerase (RNA) III (DNA directed) polypeptide A, 155kDa | POLR3A | NM_007055 | | 1.51 | 0 | 2.62E-04 |
| 8136940 | Hs.603748 | KIAA0947 | KIAA0947 | NM_015325 | | 1.54 | 0 | 0.0069 |
| 8085340 | Hs.715860 | UTP15, U3 small nucleolar ribonucleoprotein, homolog (S. cerevisiae) | UTP15 | NM_032175 | | 1.54 | 0 | 0.0135 |
| 8049963 | Hs.473317 | DEAD (Asp-Glu-Ala-Asp) box polypeptide 56 | DDX56 | NM_019082 | | 1.54 | 0 | 5.43E-04 |
| 8134745 | Hs.592283 | exostoses (multiple)-like 3 | EXTL3 | NM_001440 | | 1.55 | 0 | 0.0130 |
| 7930894 | Hs.524625 | protein arginine methyltransferase 5 | PRMT5 | NM_001039619 | | 1.56 | 0 | 0.0019 |
| 7962659 | Hs.200063 | sideroflexin 4 | SFXN4 | NM_213649 | | 1.56 | 0 | 0.0106 |
| 8095870 | Hs.724499 | nucleolar complex associated 2 homolog (S. cerevisiae) | NOC2L | NM_015658 | | 1.56 | 0 | 0.0029 |
| 7924760 | Hs.659396 // Hs.724629 | mitochondrial ribosomal protein L4 | MRPL4 | NM_146388 | | 1.57 | 0 | 0.0034 |
| 8123760 | Hs.655737 | DEAD (Asp-Glu-Ala-Asp) box polypeptide 27 | DDX27 | NM_017895 | | 1.58 | 0 | 0.0111 |
| 8140227 | Hs.647047 | chromosome 20 open reading frame 4 | C20orf4 | BC019311 | | 1.58 | 0 | 5.51E-04 |
| 7900395 | Hs.205627 | chromosome 20 open reading frame 177 | C20orf177 | NM_022106 | | 1.59 | 0 | 7.00E-04 |
| 7939738 | Hs.700338 | family with sequence similarity 98, member A | FAM98A | NM_015475 | | 1.59 | 0 | 0.0080 |
| 8013965 | Hs.654754 | mannose-6-phosphate receptor (cation dependent) | M6PR | NM_002355 | | 1.60 | 0 | 0.0044 |
| 8019988 | Hs.49774 | tocopherol (alpha) transfer protein-like | TTPAL | NM_024331 | | 1.60 | 0 | 0.0025 |
| 7911329 | Hs.453629 | glutamyl-tRNA synthetase 2, mitochondrial (putative) | EARS2 | NM_001083614 | | 1.61 | 0 | 0.0015 |
| 7997680 | Hs.461647 | interferon stimulated exonuclease gene 20kDa-like 2 | ISG20L2 | NM_030980 | | 1.62 | 0 | 0.0083 |
| 7958931 | Hs.372152 | tubulin, beta 2C | TUBB2C | NM_006088 | | 1.62 | 0 | 0.0052 |
| 8166289 | Hs.659851 | deoxynucleotidyltransferase, terminal, interacting protein 2 | DNTTIP2 | NM_014597 | | 1.62 | 0 | 0.0171 |
| 8052654 | Hs.7886 | apoptosis, caspase activation inhibitor | AVEN | NM_020371 | | 1.62 | 0 | 0.0037 |
| 8157324 | Hs.494875 | steroid 5 alpha-reductase 3 | SRD5A3 | NM_024592 | | 1.62 | 0 | 0.0140 |
| 7998117 | Hs.453629 | presenilin 2 (Alzheimer disease 4) | PSEN2 | NM_000447 | | 1.63 | 0 | 0.0132 |
| 7926037 | Hs.195471 | gem (nuclear organelle) associated protein 5 | GEMIN5 | NM_015465 | | 1.64 | 0 | 3.56E-04 |
| 8082066 | Hs.584881 | ribonuclease H1 | RNASEH1 | NM_002936 | | 1.64 | 0 | 0.0028 |
| 8007799 | Hs.654930 | moesin | MSN | NM_002444 | | 1.64 | 0 | 0.0042 |
| 7900603 | Hs.148845 | BMS1 homolog, ribosome assembly protein (yeast) | BMS1 | NM_014753 | | 1.64 | 0 | 0.0139 |
| 7915392 | Hs.648369 | trafficking protein, kinesin binding 2 | TRAK2 | NM_015049 | | 1.65 | 0 | 0.0083 |
| 7906613 | Hs.517265 | solute carrier family 25, member 44 | SLC25A44 | NM_014655 | | 1.65 | 0 | 6.31E-05 |
| 7998466 | Hs.643536 | prefoldin subunit 6 | PFDN6 | NM_001185181 | | 1.66 | 0 | 0.0096 |
| 7964089 | Hs.273397 | prefoldin subunit 6 | PFDN6 | NM_001185181 | | 1.66 | 0 | 0.0096 |
| 7949851 | Hs.382074 | prefoldin subunit 6 | PFDN6 | NM_001185181 | | 1.66 | 0 | 0.0096 |
| 8144281 | Hs.98594 | RNA pseudouridylate synthase domain containing 1 | RPUSD1 | NM_058192 | | 1.67 | 0 | 0.0061 |
| 8149521 | Hs.491143 | tissue specific transplantation antigen P35B | TSTA3 | NM_003313 | | 1.67 | 0 | 0.0154 |
| 8099581 | Hs.446201 // Hs.677572 | DEAH (Asp-Glu-Ala-His) box polypeptide 37 | DHX37 | NM_032656 | | 1.67 | 0 | 0.0110 |
| 8062190 | Hs.123159 | xylosylprotein beta 1,4-galactosyltransferase, polypeptide 7 (galactosyltransferase I) | B4GALT7 | NM_007255 | | 1.68 | 0 | 0.0048 |
| 8000791 | Hs.513491 | leucine carboxyl methyltransferase 2 | LCMT2 | NM_014793 | | 1.68 | 0 | 0.0047 |
| 7898300 | Hs.591533 | torsin family 3, member A | TOR3A | NM_022371 | | 1.71 | 0 | 0.0167 |
| 8088371 | Hs.476453 | pseudouridylate synthase 1 | PUS1 | NM_025215 | | 1.71 | 0 | 2.50E-04 |
| 7964413 | Hs.443673 | non imprinted in Prader-Willi/Angelman syndrome 2 | NIPA2 | NM_001184889 | | 1.73 | 0 | 0.0016 |
| 8031990 | Hs.453629 | bone marrow stromal cell antigen 2 | BST2 | NM_004335 | | 1.75 | 0 | 0.0032 |
| 7898070 | Hs.371823 | UDP-glucuronate decarboxylase 1 | UXS1 | NM_025076 | | 1.75 | 0 | 0.0040 |
| 8094460 | Hs.479396 | proteasome (prosome, macropain) 26S subunit, non-ATPase, 1 | PSMD1 | NM_002807 | | 1.76 | 0 | 0.0127 |
| 8061305 | Hs.187635 | uridine monophosphate synthetase | UMPS | NM_000373 | | 1.76 | 0 | 0.0042 |
| 8123606 | Hs.8162 | leucine rich repeat containing 59 | LRRC59 | NM_018509 | | 1.77 | 0 | 0.0101 |
| 8014891 | Hs.444388 | translocase of outer mitochondrial membrane 40 homolog (yeast) | TOMM40 | NM_001128917 | | 1.78 | 0 | 0.0144 |
| 8055872 | Hs.120725 | methyltransferase like 1 | METTL1 | NM_005371 | | 1.78 | 0 | 0.0050 |
| 8076387 | Hs.344088 | SET domain and mariner transposase fusion gene | SETMAR | NM_006515 | | 1.79 | 0 | 0.0062 |
| 7955142 | Hs.250712 | apolipoprotein L, 1 | APOL1 | NM_145343 | | 1.79 | 0 | 0.0069 |
| 8154333 | Hs.709425 | piggyBac transposable element derived 2 | PGBD2 | NM_170725 | | 1.80 | 0 | 0.0030 |
| 8023889 | Hs.551713 // Hs.623702 | protocadherin beta 14 | PCDHB14 | NM_018934 | | 1.81 | 0 | 0.0018 |
| 8084766 | Hs.137569 | adaptor-related protein complex 4, sigma 1 subunit | AP4S1 | NM_007077 | | 1.81 | 0 | 0.0092 |
| 7966749 | Hs.525709 | BCS1-like (S. cerevisiae) | BCS1L | NM_004328 | | 1.83 | 0 | 0.0064 |
| 7949857 | Hs.433329 | ankyrin repeat domain 36B pseudogene 1 | ANKRD36BP1 | NR_026844 | | 1.83 | 0 | 0.0187 |
| 8148501 | Hs.43666 | sterile alpha motif domain containing 9 | SAMD9 | NM_017654 | | 1.86 | 0 | 0.0044 |
| 7941087 | Hs.75199 | ceroid-lipofuscinosis, neuronal 6, late infantile, variant | CLN6 | NM_017882 | | 1.87 | 0 | 9.76E-04 |
| 8020508 | Hs.445511 | DEAH (Asp-Glu-Ala-His) box polypeptide 9 | DHX9 | NR_033302 | | 1.87 | 0 | 0.0109 |
| 8046408 | Hs.470633 | transmembrane protein 199 | TMEM199 | NM_152464 | | 1.89 | 0 | 7.39E-04 |
| 7960865 | Hs.419240 | mRNA turnover 4 homolog (S. cerevisiae) | MRTO4 | NM_016183 | | 1.90 | 0 | 0.0016 |
| 8002249 | Hs.368421 | ribosomal RNA processing 9, small subunit (SSU) processome component, homolog (yeast) | RRP9 | NM_004704 | | 1.92 | 0 | 0.0129 |
| 7960850 | Hs.655169 | HEAT repeat containing 1 | HEATR1 | NM_018072 | | 1.93 | 0 | 0.0188 |
| 8110055 | Hs.127126 | nuclear receptor coactivator 5 | NCOA5 | NM_020967 | | 1.93 | 0 | 0.0070 |
| 8140371 | Hs.488835 | FAST kinase domains 3 | FASTKD3 | NM_024091 | | 1.96 | 0 | 9.60E-04 |
| 7936856 | Hs.287537 | protein kinase, cAMP-dependent, regulatory, type I, beta | PRKAR1B | NM_002735 | | 1.97 | 0 | 0.0081 |
| 8112596 | Hs.239154 | nuclear receptor 2C2-associated protein | NR2C2AP | NM_176880 | | 1.97 | 0 | 0.0064 |
| 7921014 | Hs.314327 | NAD(P) dependent steroid dehydrogenase-like | NSDHL | NM_015922 | | 1.97 | 0 | 0.0148 |
| 7954653 | Hs.505141 | receptor (TNFRSF)-interacting serine-threonine kinase 1 | RIPK1 | NM_003804 | | 1.99 | 0 | 0.0056 |
| 8032509 | Hs.515544 | large subunit GTPase 1 homolog (S. cerevisiae) | LSG1 | NM_018385 | | 1.99 | 0 | 0.0020 |
| 7951596 | Hs.654525 | EBNA1 binding protein 2 | EBNA1BP2 | NM_006824 | | 2.01 | 0 | 0.0221 |
| 8157153 | --- | putative homeodomain transcription factor 1 | PHTF1 | NM_006608 | | 2.02 | 0 | 0.0072 |
| 8010454 | Hs.195642 | sialidase 1 (lysosomal sialidase) | NEU1 | NM_000434 | | 2.03 | 0 | 0.0077 |
| 8123080 | Hs.436977 | epithelial membrane protein 3 | EMP3 | NM_001425 | | 2.04 | 0 | 0.0042 |
| 8141305 | Hs.386324 | MPV17 mitochondrial membrane protein-like 2 | MPV17L2 | NM_032683 | | 2.05 | 0 | 0.0073 |
| 8085537 | Hs.475565 // Hs.706478 | DPH2 homolog (S. cerevisiae) | DPH2 | NM_001384 | | 2.06 | 0 | 0.0027 |
| 8129924 | Hs.535820 | torsin family 1, member A (torsin A) | TOR1A | NM_000113 | | 2.07 | 0 | 0.0038 |
| 7920341 | Hs.4779 | neuroplastin | NPTN | NM_012428 | | 2.13 | 0 | 0.0063 |
| 8035187 | Hs.651587 | testis expressed 261 | TEX261 | NM_144582 | | 2.14 | 0 | 0.0029 |
| 7955589 | Hs.524430 | cytochrome b5 reductase 1 | CYB5R1 | NM_016243 | | 2.15 | 0 | 0.0014 |
| 7923753 | Hs.497512 | processing of precursor 1, ribonuclease P/MRP subunit (S. cerevisiae) | POP1 | NM_001145860 | | 2.15 | 0 | 0.0015 |
| 8156506 | Hs.437943 | solute carrier family 4, sodium bicarbonate cotransporter, member 4 | SLC4A4 | NM_001098484 | | 2.17 | 0 | 0.0183 |
| 8058670 | Hs.604950 | FtsJ homolog 3 (E. coli) | FTSJ3 | NM_017647 | | 2.23 | 0 | 0.0212 |
| 8049961 | Hs.438454 | cytochrome P450, family 20, subfamily A, polypeptide 1 | CYP20A1 | NM_177538 | | 2.23 | 0 | 0.0034 |
| 8028744 | Hs.456377 | Der1-like domain family, member 2 | DERL2 | NM_016041 | | 2.23 | 0 | 0.0215 |
| 8012349 | Hs.445534 | spinster homolog 1 (Drosophila) | SPNS1 | NM_032038 | | 2.28 | 0 | 0.0062 |
| 7987960 | Hs.724640 | methyltransferase like 13 | METTL13 | NM_015935 | | 2.28 | 0 | 0.0043 |
| 7949503 | Hs.719933 | spastic ataxia of Charlevoix-Saguenay (sacsin) | SACS | NM_014363 | | 2.28 | 0 | 0.0107 |
| 8119161 | Hs.81170 | cysteine rich transmembrane BMP regulator 1 (chordin-like) | CRIM1 | NM_016441 | | 2.30 | 0 | 0.0256 |
| 7979611 | Hs.654571 | transmembrane and coiled-coil domains 1 | TMCO1 | NM_019026 | | 2.30 | 0 | 0.0036 |
| 7923662 | Hs.497487 | dolichol kinase | DOLK | NM_014908 | | 2.31 | 0 | 0.0070 |
| 8116548 | Hs.29106 | pannexin 1 | PANX1 | NM_015368 | | 2.33 | 0 | 0.0039 |
| 8119088 | Hs.370771 | mitochondrial E3 ubiquitin protein ligase 1 | MUL1 | NM_024544 | | 2.38 | 0 | 0.0103 |
| 7951752 | Hs.503891 | heat shock 105kDa/110kDa protein 1 | HSPH1 | NM_006644 | | 2.42 | 0 | 0.0017 |
| 8062623 | Hs.268177 | G elongation factor, mitochondrial 1 | GFM1 | NM_024996 | | 2.43 | 0 | 0.0074 |
| 7997626 | Hs.578546 | cytochrome b-245, beta polypeptide | CYBB | NM_000397 | | 2.45 | 0 | 0.0091 |
| 8036956 | Hs.585105 | protein O-glucosyltransferase 1 | POGLUT1 | NM_152305 | | 2.56 | 0 | 0.0026 |
| 7899394 | Hs.10649 | kynurenine 3-monooxygenase (kynurenine 3-hydroxylase) | KMO | NM_003679 | | 2.69 | 0 | 0.0088 |
| 8113981 | Hs.519568 | G protein-coupled receptor 172A | GPR172A | NM_024531 | | 2.73 | 0 | 0.0022 |
| 8047577 | Hs.444982 | claudin 12 | CLDN12 | NM_001185072 | | 2.73 | 0 | 0.0089 |
| 7953981 | Hs.504765 | major facilitator superfamily domain containing 5 | MFSD5 | NM_001170790 | | 2.75 | 0 | 0.0217 |
| 7961798 | Hs.657542 | arylsulfatase B | ARSB | NM_000046 | | 2.82 | 0 | 0.0029 |
| 8066393 | Hs.441737 | lectin, mannose-binding 2-like | LMAN2L | NM_001142292 | | 2.85 | 0 | 0.0063 |
| 8067248 | Hs.473179 | TM2 domain containing 2 | TM2D2 | NM_031940 | | 2.88 | 0 | 0.0082 |
| 7945342 | Hs.453629 | cystinosis, nephropathic | CTNS | NM_004937 | | 2.92 | 0 | 0.0030 |
| 7957260 | Hs.205558 | SLAM family member 7 | SLAMF7 | NM_021181 | | 3.02 | 0 | 5.26E-05 |
| 8034151 | Hs.591002 | UTP20, small subunit (SSU) processome component, homolog (yeast) | UTP20 | NM_014503 | | 3.02 | 0 | 0.0051 |
| 7996807 | Hs.461074 // Hs.709017 | guanylate binding protein 1, interferon-inducible, 67kDa | GBP1 | NM_002053 | | 3.34 | 0 | 0.0198 |
| 8064302 | Hs.473317 | glycerol-3-phosphate acyltransferase, mitochondrial | GPAM | NM_020918 | | 3.35 | 0 | 0.0164 |
| 7988286 | Hs.656271 // Hs.683876 | cytochrome P450, family 4, subfamily F, polypeptide 11 | CYP4F11 | NM_021187 | | 3.44 | 0 | 0.0037 |
| 7914433 | Hs.524138 | interleukin 7 receptor | IL7R | NM_002185 | | 3.45 | 0 | 0.0225 |
| 8025945 | Hs.675132 | Fc receptor-like 4 | FCRL4 | NM_031282 | | 3.55 | 0 | 0.0212 |
| 8087447 | Hs.349110 // Hs.512587 | phosphatidylinositol glycan anchor biosynthesis, class W | PIGW | NM_178517 | | 3.57 | 0 | 0.0028 |
| 7913864 | Hs.209983 | transmembrane protein 229B | TMEM229B | NM_182526 | | 3.59 | 0 | 0.0041 |
| 8005765 | Hs.446017 | solute carrier organic anion transporter family, member 2B1 | SLCO2B1 | NM_001145211 | | 5.87 | 0 | 9.33E-04 |
| 8156393 | Hs.88417 | core-binding factor, runt domain, alpha subunit 2; translocated to, 3 | CBFA2T3 | NM_005187 | | 6.46 | 0 | 0.0240 |
| 8129193 | Hs.656959 | GLI pathogenesis-related 1 | GLIPR1 | NM_006851 | | 6.62 | 0 | 0.0039 |
