# Supplementary material for: The Transcriptional Co-Repressor Myeloid Translocation Gene 16 Inhibits Glycolysis and Stimulates Mitochondrial Respiration
Source: PLoS One. 2013 Jul 1;8(7):e68502. doi: 10.1371/journal.pone.0068502 (PMC3698176; doi:10.1371/journal.pone.0068502)
Supplement: Table S2 — Gene expression changes upon MTG16 expression in Raji/MTG16 Tet-On 3G cells. The list shows genes that were downregulated at least 0.8-fold or upregulated at least 1.3-fold with the average fold change between three biological replicate microarrays depicted as a ratio to 1. (DOC) [file pone.0068502.s005.doc]

| **Gene Name** | **mRNA Accession** | **Primer sequences** | **Gene Functions** |
| --- | --- | --- | --- |
| 6-phosphofructo-2-kinase/fructose-2,6-biphosphatase 3  (PFKFB3) | NM_004566 | Hs00998700_m1  (Taqman Assay, life technologies) | Regulates steady state level of fructose-2,6-bisphosphate, an allosteric activator of key glycolytic enzyme, phosphofructokinase |
| 6-phosphofructo-2-kinase/fructose-2,6-biphosphatase 4  (PFKFB4) | NM_004567 | Hs00190096_m1  (Taqman Assay, life technologies) | Regulates steady state level of fructose-2,6-bisphosphate, an allosteric activator of key glycolytic enzyme, phosphofructokinase |
| Pyruvate dehydrogenase kinase, isozyme 1  (PDK1) | NM_002610 | Hs01561850_m1  (Taqman Assay, life technologies) | Phosphorylates and deactivates pyruvate dehydrogenase, thus blocking oxidative phosphorylation. |
| Pim-2 oncogene  (Pim2) | NM_006875 | Hs00179139_m1  (Taqman Assay, life technologies) | Proto-oncogene with serine/therionine kinase activity involved in cell survival and cell proliferation. It excerts its activity through regulation of MYC transcriptional activity and regulation of progression of cell cycle. |
| Hairy and enhancer of split 1, (Drosophila) (HES1) | NM_005524 | Forward 5’TCTGGAAATGACAGTGAAGCAC3’  Reverse  5’GTCACCTCGTTCATGCACTC3’ | Member of HES gene family, which encode nuclear protein that suppresses transcription. It plays an important role in Notch signaling pathway. |
| B-cell CLL/lymphoma 6  (BCL6) | NM_001706 | Forward 5’AGAAGCCCTATCCCTGTGAAAT3’  Reverse 5’GACGGAAATGCAGGTTACACTT3’ | Acts as sequence specific repressor of transcription. It has shown to be translocated and hypermutated in diffuse large B-cell Lymphoma. Interact with several repressors to inhibit repression. |
| Adenosine deaminase  (ADA) | NM_000022 | Forward 5’ACATGGGCTTTACTGAAGAGGA3’  Reverse 5’GGCTTTATAGAGCAGGTCGAGA3’ | Catalyzes the hydrolytic deamination of adenosine and 2-deoxynosine. Plays an important role in purine metabolism and in adenosine homeostasis. Modulates signaling by extracellular adenosine, and so contributes indirectly to cellular signaling events. |
| Cyclin-dependent kinase 18  (CDK18) | NM_212503 | Forward 5’CTTCTCTGAGTTCCGCACCTAC3’  Reverse  5’CTGACATGCGACTCTTGGATT3’ | Serine/therionine kinase, may play a role in signal transduction pathway in differentiated cells. Has been shown to be involved in breast cancer. |
| Cyclin G2  (CCNG2) | NM_004354 | Forward 5’ATAGTGTTCCTGAGCTGCCAAC3’  Reverse  5’GAGCTGCTGAGACTCTCCTCTC3’ | Belong to family of cycline which is regulator of CDK kinases which promotes cell cycle (G1/S and G2/M) transition. |
| Huntingtin interacting protein 1  (HIP1) | NM_005338 | Forward 5’ATGACGCTGACACAGATCAAAC3’  Reverse  5’AGCAAGCTCGTAGTGCTTTTTC3’ | Membrane associated protein colocalizes with huntingtin. Thought to play role in filamentous network. Has been implicated in hematologic pathogenesis. |
| Pyruvate kinase, muscle  (PKM2) | NM_182470 | Forward 5’GCAGCAGCTTTGATAGTTCTGA3’  Reverse 5’GAAGATGCCACGGTACAGGT3’ | Glycolytic enzyme that catalyzes the transfer of a phosphoryl group from phosphoenolpyruvate (PEP) to ADP,generating ATP. The transition between the 2 forms contributes to the control of glycolysis and is important for tumor cell proliferation and survival. |

Table S2. Selected candidate genes, mRNA accession numbers, primer/probe sequences, and functions.
